# Exploratory quantitative EEG characteristics in children with autism spectrum disorder

**DOI:** 10.3389/fpsyt.2025.1689000

**Published:** 2025-11-05

**Authors:** Marta Kopańska, Danuta Ochojska, Izabela Sarzyńska, Julia Trojniak, Jacek Szczygielski

**Affiliations:** ^1^ Department of Medical Psychology, Faculty of Medicine, Collegium Medicum, University of Rzeszow, Rzeszow, Poland; ^2^ Laboratory for Clinical Neurophysiology, Clinimetry and Microsurgical Training, Centre for Innovative Research in Medical Sciences, University of Rzeszów, Rzeszow, Poland; ^3^ Department of Psychology, Faculty of Health Sciences and Psychology, University of Rzeszow, Rzeszow, Poland; ^4^ Students Science Club “Reh-Tech”, Institute of Medical Sciences, University of Rzeszow, Rzeszow, Poland; ^5^ Department of Neurosurgery, Faculty of Medicine, University of Rzeszow, Rzeszow, Poland; ^6^ Department of Neurosurgery, Faculty of Medicine, Saarland University, Homburg, Germany

**Keywords:** autism spectrum disorders, ASD, brainwave model, quantitative electroencephalography, qEEG

## Abstract

**Introduction:**

Autism Spectrum Disorders (ASD) are currently one of the most common childhood conditions. It is estimated that they affect approximately 1 in 31 children. Early and rapid diagnosis can increase a child’s chances of reaching full developmental, social, and educational potential despite their condition.

**Methods:**

Our study aimed to describe a brainwave pattern in children with mild autism spectrum disorder (Level-1 according to DSM-5) based on quantitative electroencephalography (QEEG) analysis. The QEEG study is one of the valuable electrophysiological methods used in neurology and psychiatry, becoming more and more popular for diagnosing ASD. Our study included 48 children aged 7–10 years. Based on previous clinical examinations, 24 of them were diagnosed with mild ASD (mASD). Quantitative electroencephalography for Delta, Theta, Alpha, sensorimotor rhythm (SMR), Beta1, and Beta2 waves was performed using electrodes placed at thirteen recording points (frontal: FzF3F4, central: CzC3C4, parietal: P3PzP4, temporal: T3T4, and occipital: O1O2 points) with eyes open and closed.

**Results:**

A comparison of the results between the mASD group and control group revealed significantly higher amplitude values for all Delta, Theta, Alpha, SMR, Beta1, and Beta2 wave measurements in the mASD population. Furthermore, the overrepresentation of Beta2-waves could be discerned in mASD children, as compared to their non-ASD-affected peers.

**Discussion:**

The described pattern may help screen for mASD or confirm the diagnosis in the pediatric population of mASD-suspected patients. Additionally, it is worth noting that the results obtained demonstrate the importance of QEEG in detecting different patterns of brain activity in children with ASD, which plays a significant role in better understanding the heterogeneity of this disorder.

## 1 Introduction

In 1943, child psychiatrist Leo Kanner first characterized autism as an innate barrier to forming normal, biologically coded emotional interactions with others (1). Among the disorders that relate to this definition, autism and Autism Spectrum Disorders (ASD) are currently distinguished. According to the most recent CDC report, approximately 1 in 31 children (3.2%) aged 8 years have been diagnosed with ASD, representing a nearly threefold increase compared to prevalence rates reported in the early 2000s (2, 3). On a global scale, around 1% of children are affected by ASD (2), and it occurs about four times more often in boys than in girls (4–6). Autism and ASD are now viewed as a collection of neurodevelopmental conditions, some of which can be attributed to specific etiological factors, such as Mendelian single-gene mutations. However, most cases likely arise from complex interactions between genetic and non-genetic risk factors (7). The heritability of this disorder is estimated to be between 70% and 90% (4). Many forms are commonly defined by specific behaviors, focusing on atypical development in social communication and highly restricted or repetitive behaviors and interests (7). ASD manifests as difficulties in social communication, characterized by persistent deficits in daily communication and repetitive patterns of behavior, interests, or activities (8).

Approximately 25% of children with autism and ASD are diagnosed between the ages of 2 and 3, with around 30% experiencing skill regression. About 60% of individuals with ASD show intellectual limitations in early childhood. Individuals with ASD can lead fulfilling lives, but a delayed diagnosis can lead to a reduced quality of life. Early detection of ASD can be crucial for starting interventions and therapies more early enough, potentially reducing the severity of the symptoms (8).

In our study, we employed quantitative electroencephalography as a modern diagnostic tool, assessing objectively the electrophysiological activity of the brain (9–11). This method was chosen for its relative ease of use and efficiency compared to other functional brain imaging techniques (12). It offers high reliability and allows for cost-effective testing, which is an additional advantage (11). QEEG involves the quantitative analysis of EEG recordings using statistical signal processing (13). Unlike standard EEG, QEEG facilitates more straightforward interpretation due to better visual presentation of its results (14).Notably, QEEG is a highly sensitive test capable of detecting even subtle changes in cortical function (15). Quantitative electroencephalography is a frequently used method for diagnosing neurological disorders. QEEG involves computer-based measurement and analysis of EEG data. The use of digital techniques enables rapid analysis of the recorded signals and graphical representation of the brain’s bioelectrical activity (16). With QEEG, the following frequency bands can be studied: Delta (0.5–4 Hz), Theta (4–8 Hz), Alpha (8–12 Hz), Sensorimotor Rhythm (SMR) (12–15 Hz), Beta1 (15–20 Hz) and Beta2 (20–34 Hz) (17). Additionally, QEEG allows for the detection of abnormalities in cortical function and demonstrates the correlation between disorders and power maps, making it an additional asset of this method (18). Currently, many researchers recognize that QEEG may also be helpful in the diagnostic workup of ASD.

In the context of changes seen in children with autism and ASD, the percentage distribution norms of brainwaves in children according to Sterman are highly significant — **Table 1**.

**Table 1:** Percentage of brainwaves in healthy children – developed according to Sterman (19).

Wave	Percentage of brainwaves [%]
Delta	~29
Theta	~22
Alpha	~18
SMR	~13
Beta 1	~9
Beta 2	~9

It is worth noting that EEG studies in individuals with ASD have revealed abnormalities in brain bioelectrical activity, particularly in theta and beta waves (20–23). Changes have also been described in the delta, alpha, and gamma bands (24). Other authors point to increased heterogeneity in QEEG recordings in individuals with ASD (25). Therefore, in our study, we focused on analyzing delta, theta, alpha, SMR, beta1 and beta2 waves, i.e. the bands most often described as disturbed and functionally significant in ASD. Delta waves are believed to play an important role in the formation of slow waves associated with attention and the detection of relevant stimuli. Theta waves, on the other hand, are associated with memory processes. Alpha waves are associated with sensory processing and cortical excitability, while beta waves are associated with cognitive processes and emotional control. Our analysis also included the sensorimotor rhythm (SMR), which is associated with motor regulation and inhibition control, often described as atypical in ASD (24, 26, 27).

Given previous scientific reports indicating the presence of abnormal neurodevelopmental processes leading to atypical brain structure, activity, and changes in structural and functional neural interconnections in individuals with ASD, our study was focused on brainwave analysis in patients with ASD as the potential electrophysiologic representation of these abnormal brain properties. Our primary hypothesis was that QEEG records of pediatric mASD subjects differ from those of non-affected children and that the pattern of expected abnormalities in brainwave frequency bands, such as Delta, Theta, Alpha, SMR, Beta1, and Beta2, and their deviations from normative values will be characteristic enough to constitute a neurophysiological biomarker for mASD. To date, very few studies have been conducted to analyze QEEG results in the pediatric ASD population, which encouraged us to provide our own analysis in this area. It should be emphasized, however, that the present study is exploratory in nature. Its aim is the preliminary identification of potential neurophysiological biomarkers that may provide a basis for future, larger-scale, and more detailed research in this field.

## 2 Materials and methods

The study was conducted on a group of 48 children aged 7–10 years, residenting in Poland, the Subcarpathian region. Participants for the mASD group were recruited from patients of the Psychological-Pedagogical Counseling Center in Rzeszow, following their clinical diagnosis. The control group consisted of typically developing children recruited via advertisements posted in local schools and community centers within a region of a similar socio-economic status. The main group included 24 participants (11 girls and 13 boys) diagnosed with ASD – Level -1 (mild symptoms). These children had an opinion from psychological counselling center (disability degree certificate).

This diagnosis was confirmed using a multi-step diagnostic protocol, including an assessment based on the Autism Diagnostic Observation Schedule (ADOS-2), in the Polish adaptation and validation by Chojnicka and Pisula (28). Thus, the diagnostic procedures applied in this study relied on the officially adapted and psychometrically validated Polish version of ADOS-2, ensuring reliability and reproducibility in the local clinical context. Informed consent was obtained from all parents or legal guardians of participants involved in this study. The study was approved by the Ethics Committee of Rzeszow University - number of permission 021/05/2024 and 043/06/2025 and all methods were performed in accordance with the Declaration of Helsinki and relevant institutional and national guidelines and regulations. Clinical diagnostics (DSM-5/ADOS) were performed in specialized centers outside the study protocol; therefore, raw test scores are not reported here, in line with the ethics approval.

All children in the ASD group underwent a detailed speech and language assessment to evaluate the degree of delay and impairment in speech, language, and communication skills. These domains are critical, as they often reflect the level of dysfunction in ASD and can vary significantly between individuals. The final clinical diagnosis was established by a specialist physician, who synthesized the results of the conducted tests and direct observation of each child. Only children without co-occurring neurological conditions, such as epilepsy, genetic disorders (e.g., Fragile X syndrome, Rett syndrome), or other neurodevelopmental disorders, were included in the study. This ensured that the analysis focused solely on the symptomatology characteristic of ASD.

The study group consisted of participants diagnosed with mild ASD (according to DSM-5: Level 1- (requiring support; noticeable difficulties with social communication and interaction), reflecting a specific subset of the autism spectrum. Focusing our study on mild forms of ASD allowed the detailed analysis of QEEG requiring certain degree of subjects’ cooperation during the recording. Despite being categorized as Level-1 symptoms of ASD, the children in this group still presented with a range of cognitive, communicative, and behavioral profiles, highlighting the individual variability even within a milder presentation of the disorder.

The study also included a control group of typically developing children (10 girls and 14 boys), matched in age (7–10 years) and socio-economic background. Children were randomly selected for this group. Control group participants showed no symptoms related to ASD or other neurodevelopmental disorders, according to DSM-5 criteria. Additionally, family histories were reviewed to exclude cases of autism or other significant neuropsychiatric conditions among immediate family members, thus minimizing potential genetic influences on the study outcomes – **Table 2**.

**Table 2:** Inclusion and exclusion criteria for participants in the ASD group.

Inclusion criteria	Exclusion criteria
1. Age of participants between 7 and 10 years 2. Diagnosis of mild Autism Spectrum Disorder established according to DSM-5 criteria (Level-1 requiring support) 3. Confirmation of mild ASD diagnosis using the Autism Diagnostic Observation Schedule (ADOS-2) 4. Absence of co-occurring neurological disorders 5. A range of functioning levels allowing for a comprehensive analysis of the spectrum of ASD symptoms	1. Presence of other neurological disorders or genetic conditions 2. Presence of other neurodevelopmental disorders 3. Family history of autism or other significant neuropsychiatric disorders in the control group, to eliminate potential genetic influences 4. Lack of parental or guardian consent for the child’s participation in the study

Before conducting inter-group comparisons, the distribution of data for each variable was assessed for normality using the Shapiro-Wilk test. The test results indicated that for most analyzed variables, the data distribution in at least one of the groups significantly deviated from a normal distribution (p<0.05). Due to the violation of the normality assumption, the non-parametric Mann-Whitney U test was employed to compare differences in brainwave amplitudes between the ASD group and the control group. The statistical significance level was set at p<0.05. Detailed results of normality tests are shown in **Table 3**.

**Table 3:** Shapiro-Wilk test results for normality of brainwave amplitude distribution in both study groups (eyes open and eyes closed).

Wave	Group	Fz	F3	F4	Cz	C3	C4	P3	P4
Delta, open eyes	Control group	p<0.001	p<0.001	p=0.001	p<0.001	p<0.001	p<0.001	p=0.003	p=0.001
	Study group	p=0.029	p=0.012	p=0.056	p=0.014	p<0.001	p=0.018	p=0.006	p=0.002
Theta, open eyes	Control group	p<0.001	p<0.001	p<0.001	p<0.001	p<0.001	p<0.001	p<0.001	p<0.001
	Study group	p=0.004	p=0.018	p<0.001	p=0.037	p=0.041	p=0.058	p=0.078	p=0.093
Alpha, open eyes	Control group	p=0.001	p=0.001	p<0.001	p=0.001	p=0.001	p<0.001	p=0.001	p<0.001
	Study group	p=0.005	p=0.008	p=0.371	p=0.002	p=0.028	p=0.004	p=0.011	p=0.001
SMR, open eyes	Control group	p=0.002	p<0.001	p<0.001	p=0.003	p<0.001	p<0.001	p<0.001	p=0.001
	Study group	p=0.001	p=0.363	p=0.054	p=0.002	p=0.189	p=0.006	p=0.097	p=0.019
Beta1, open eyes	Control group	p=0.001	p=0.003	p<0.001	p<0.001	p<0.001	p<0.001	p=0.001	p=0.001
	Study group	p=0.21	p=0.009	p=0.055	p=0.251	p=0.008	p=0.084	p=0.073	p=0.003
Beta2, open eyes	Control group	p<0.001	p<0.001	p<0.001	p<0.001	p<0.001	p<0.001	p<0.001	p<0.001
	Study group	p=0.064	p=0.005	p=0.001	p=0.023	p=0.103	p=0.044	p=0.018	p=0.05
Delta, closed eyes	Control group	p<0.001	p<0.001	p=0.001	p<0.001	p<0.001	p<0.001	p=0.003	p=0.001
	Study group	p=0.002	p=0.02	p=0.006	p=0.083	p=0.064	p=0.127	p=0.019	p=0.011
Theta, closed eyes	Control group	p<0.001	p<0.001	p<0.001	p<0.001	p<0.001	p<0.001	p<0.001	p<0.001
	Study group	p=0.317	p=0.227	p=0.193	p=0.162	p=0.237	p=0.352	p=0.004	p=0.033
Alpha, closed eyes	Control group	p=0.001	p=0.001	p<0.001	p=0.001	p=0.001	p<0.001	p=0.001	p<0.001
	Study group	p=0.072	p=0.092	p=0.215	p=0.036	p=0.075	p=0.259	p=0.185	p=0.025
SMR, closed eyes	Control group	p=0.002	p<0.001	p<0.001	p=0.003	p<0.001	p<0.001	p<0.001	p=0.001
	Study group	p<0.001	p=0.002	p=0.008	p=0.001	p=0.003	p=0.008	p=0.003	p=0.553
Beta1, closed eyes	Control group	p=0.001	p=0.003	p<0.001	p<0.001	p<0.001	p<0.001	p=0.001	p=0.001
	Study group	p=0.017	p=0.089	p=0.265	p=0.051	p=0.34	p=0.017	p=0.028	p=0.062
Beta2, closed eyes	Control group	p<0.001	p<0.001	p<0.001	p<0.001	p<0.001	p<0.001	p<0.001	p<0.001
	Study group	p=0.047	p=0.004	p=0.004	p=0.157	p=0.268	p=0.332	p=0.005	p=0.271

### 2.1 QEEG procedures

#### 2.1.1 Electrode positioning and recording conditions

Patients were then subjected to QEEG according to clinical standards, which provided a stable baseline of brainwave amplitudes for subsequent analysis. Electroencephalogram recordings were acquired using the DigiTrack 15 system (Elmiko, Poland), which meets clinical and research requirements in accordance with PN-EN 60601 and CE standards.

A 13-electrode setup, as QEEG standard derived from the international 10–20 system, was employed. This included central (Cz, C3, C4), frontal (Fz, F3, F4), parietal (P3, Pz, P4), temporal (T3, T4), and occipital (O1, O2) points. Ear electrodes A1–A2 served as the reference or neutral electrodes (depending on the averaging schema), adhering to quantitative EEG analysis standards. These specific locations were chosen due to their established significance in processing cognitive, social, and sensorimotor functions, which are often impaired in individuals with ASD (17). This simplified setup also offered the advantage of minimizing patient preparation time, a crucial factor in maintaining cooperation and reducing stress levels in children with ASD, thereby facilitating a more effective and comfortable experience of participation in the experiment.

The recordings were made at rest in two states: with eyes closed (EC) and eyes open (EO). Each state lasted 3 minutes and was performed in the same order (EC, then EO). Participants were instructed to remain still, minimize muscle activity, and avoid blinking or other movements that could generate artefacts. The children received simple verbal instructions and brief reminders when necessary; a parent was also present in the room to facilitate cooperation. Segments disturbed by movement or lack of cooperation were excluded, but sufficient valid data were obtained from all participants. The assessment took place in a quiet, moderately lit room, with electrode impedance maintained below 5 kΩ.

### 2.2 Technical parameters and signal processing

The technical parameters for data acquisition were as follows:

- Sampling frequency: 500 Hz.
- High-pass filter: 0.5 Hz.
- Low-pass filter: 70 Hz.
- Notch filter: 50 Hz (for elimination of power line interference).
- A/D converter resolution: 24 bits.

The EEG signal was continuously monitored. For artifact removal, initial preprocessing involved semi-automatic algorithms within the DigiTrack system, followed by manual inspection by an experienced EEG analyst. No additional cleaning techniques, such as Independent Component Analysis (ICA), were applied unless otherwise specified.

The EEG signal was then transformed. Spectrum analysis, typically performed using the Fast Fourier Transform (FFT) algorithm [f(z)=A(z)+j*F(z)], yielded peak-to-peak amplitude results in the DigiTrack FFT panel, allowing for direct comparison with existing literature.

### 2.3 Quantitative EEG analysis

The EEG data underwent QEEG performed by a researcher awarded the Board Certification in EEG Biofeedback. The following parameters were calculated:

- Mean wave amplitudes (in µV).
- Percentage contributions of frequency bands relative to total EEG activity.

The following frequency bands were analyzed:

- Delta (0.5–3 Hz).
- Theta (4–8 Hz).
- Alpha (8–12 Hz).
- Sensorimotor Rhythm (SMR) (12–15 Hz).
- Beta1 (15–20 Hz).
- Beta2 (20–34 Hz) (17).

## 3 Results

### 3.1 Delta – eyes-open

**Figure 1:**
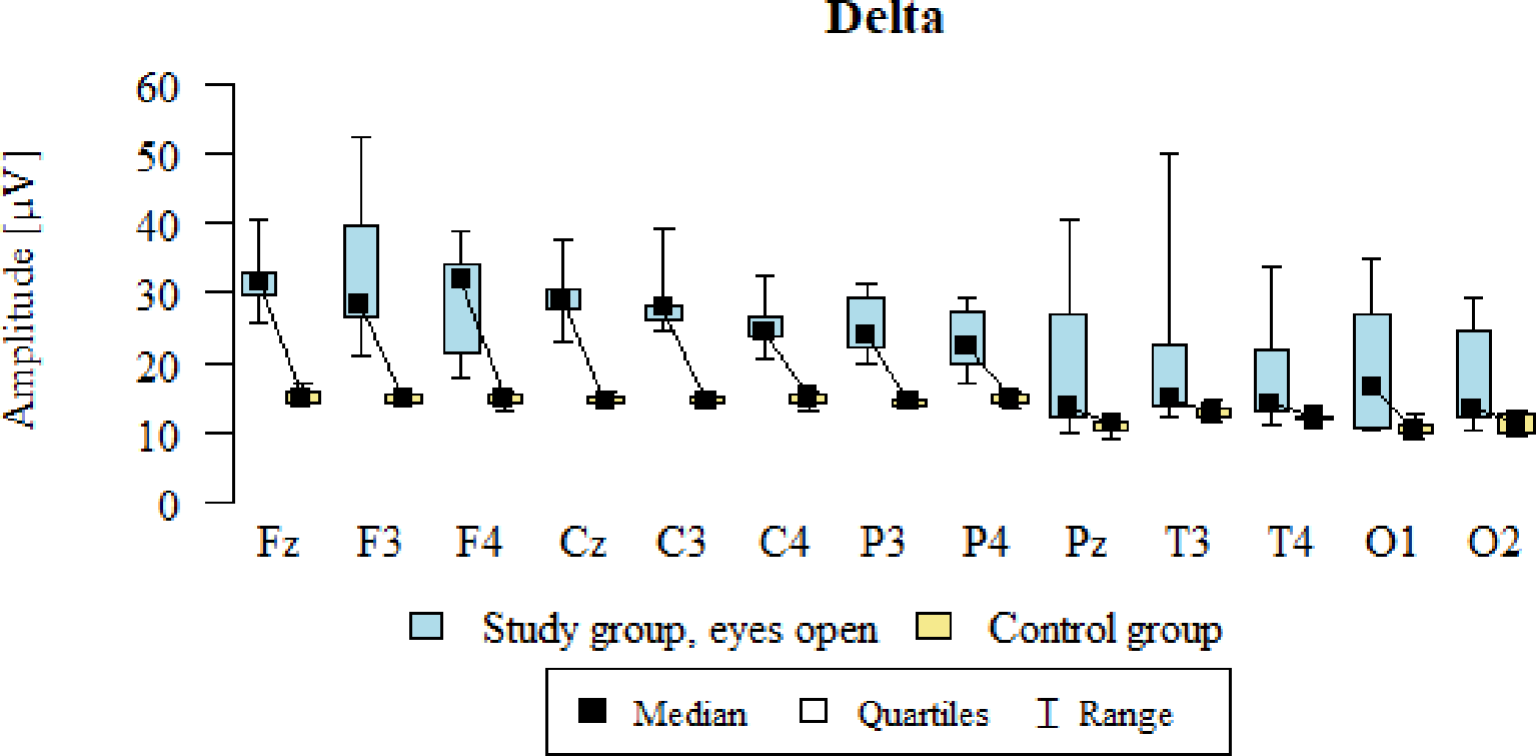
Delta wave amplitude (µV) in the eyes-open condition for the ASD group (blue) and control group (yellow).

Values of p < 0.05 indicate statistically significant differences. In the eyes-open condition, Delta wave amplitude was significantly higher in all frontal (Fz, F3, F4), central (Cz, C3, C4), parietal (P3, Pz, P4), temporal (T3, T4), and occipital (O1, O2) sites in the ASD group compared to controls (**Table 4**; **Figure 1**).

**Table 4:** Results of Delta wave study (waves from channels Fz, F3, F4, Cz, C3, C4, P3, Pz, P4, T3, T4, O1, O2) with eyes open.

Parameter	Group	N	Mean	SD	Median	Min	Max	Q1	Q3	p
Fz	Study group, eyes open	24	31.53	3.83	31.66	25.8	40.68	29.85	33.17	p<0.001 *
	Control group	24	15.3	1.09	15	14.23	16.98	14.48	15.82	
F3	Study group, eyes open	24	33.03	10.55	28.26	21.11	52.61	26.44	39.8	p<0.001 *
	Control group	24	15.05	0.75	15	14.23	15.98	14.38	15.66	
F4	Study group, eyes open	24	28.92	7.26	31.85	17.78	38.95	21.5	34.1	p<0.001 *
	Control group	24	14.82	1.03	15.01	13.28	15.98	14.29	15.54	
Cz	Study group, eyes open	24	29.42	4.06	29.12	23.14	37.83	27.97	30.48	p<0.001 *
	Control group	24	14.8	0.71	14.5	14.23	15.98	14.38	14.91	
C3	Study group, eyes open	24	28.21	3.78	27.96	24.48	39.43	26.05	28.39	p<0.001 *
	Control group	24	14.8	0.71	14.5	14.23	15.98	14.38	14.91	
C4	Study group, eyes open	24	25.43	3.52	24.37	20.67	32.55	23.75	26.82	p<0.001 *
	Control group	24	14.87	1.06	15.11	13.28	15.98	14.29	15.69	
P3	Study group, eyes open	24	25.29	4.05	23.95	19.7	31.39	22.25	29.32	p<0.001 *
	Control group	24	14.43	0.7	14.5	13.43	15.29	14.03	14.89	
P4	Study group, eyes open	24	23.53	4.36	22.37	17.17	29.55	19.98	27.54	p<0.001 *
	Control group	24	14.89	0.97	15.05	13.48	15.98	14.34	15.6	
Pz	Study group, eyes open	24	19.69	10.13	13.72	9.93	40.68	12.32	27.03	p<0.001 *
	Control group	24	11.04	0.87	11.36	9.33	12.11	10.41	11.64	
T3	Study group, eyes open	24	20.26	10.96	14.99	12.14	49.94	13.76	22.5	p<0.001 *
	Control group	24	12.88	0.92	12.71	11.5	14.62	12.18	13.5	
T4	Study group, eyes open	24	18.05	7.29	14.26	11.3	33.82	13.07	21.67	p=0.001 *
	Control group	24	12.29	0.95	12.15	10.77	14.11	11.84	12.41	
O1	Study group, eyes open	24	19.17	9.06	16.64	10.25	34.83	10.69	27.16	p<0.001 *
	Control group	24	10.58	1.13	10.37	9.04	12.83	9.94	10.95	
O2	Study group, eyes open	24	17.81	7.13	13.45	10.24	29.43	12.28	24.66	p<0.001 *
	Control group	24	11.33	1.32	11.46	9.59	12.93	9.95	12.73	

p - Mann-Whitney test, SD - standard deviation, Q1 - lower quartile, Q3 - upper quartile.

*statistically significant (p<0.05).Values represent the wave amplitude (in µV) with key distribution parameters (including median and quartiles).

### 3.2 Theta – eyes-open (EO)

**Figure 2:**
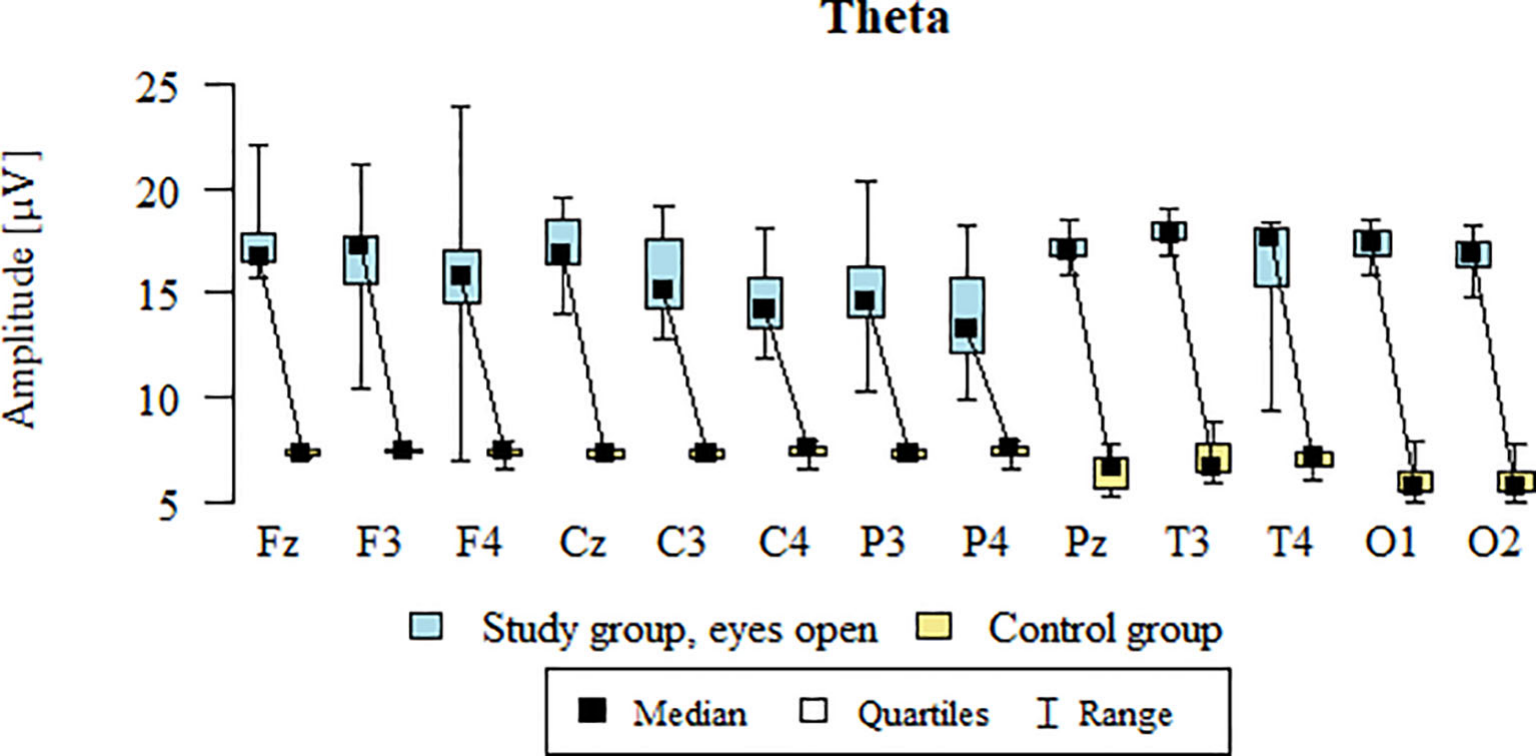
Theta wave amplitude (µV) in the eyes-open condition for the ASD group (blue) and control group (yellow).

Values of p < 0.05 indicate statistically significant differences. In the eyes-open condition, Theta wave amplitude was significantly higher in all frontal (Fz, F3, F4), central (Cz, C3, C4), parietal (P3, Pz, P4), temporal (T3, T4), and occipital (O1, O2) sites in the ASD group compared to controls (**Table 5**; **Figure 2**).

**Table 5:** Results of Theta wave study (waves from channels Fz, F3, F4, Cz, C3, C4, P3, Pz, P4, T3, T4, O1, O2) with eyes open.

Parameter	Group	N	Mean	SD	Median	Min	Max	Q1	Q3	p
Fz	Study group, eyes open	24	17.58	1.78	16.74	15.79	22.1	16.51	17.85	p<0.001 *
	Control group	24	7.36	0.16	7.35	7.18	7.54	7.2	7.5	
F3	Study group, eyes open	24	16.43	3.28	17.26	10.41	21.2	15.51	17.76	p<0.001 *
	Control group	24	7.41	0.13	7.44	7.21	7.54	7.34	7.5	
F4	Study group, eyes open	24	15.5	4.5	15.75	7.02	23.95	14.47	17.09	p<0.001 *
	Control group	24	7.34	0.5	7.46	6.54	7.89	7.22	7.58	
Cz	Study group, eyes open	24	17.22	1.73	16.86	14.05	19.55	16.4	18.49	p<0.001 *
	Control group	24	7.33	0.2	7.35	7.08	7.54	7.18	7.5	
C3	Study group, eyes open	24	15.78	2.09	15.18	12.86	19.19	14.26	17.59	p<0.001 *
	Control group	24	7.33	0.2	7.35	7.08	7.54	7.18	7.5	
C4	Study group, eyes open	24	14.52	1.81	14.18	11.93	18.07	13.41	15.66	p<0.001 *
	Control group	24	7.38	0.52	7.55	6.54	7.89	7.22	7.71	
P3	Study group, eyes open	24	15.09	2.57	14.64	10.25	20.39	13.94	16.27	p<0.001 *
	Control group	24	7.33	0.2	7.35	7.08	7.54	7.18	7.5	
P4	Study group, eyes open	24	13.78	2.48	13.23	9.92	18.19	12.16	15.77	p<0.001 *
	Control group	24	7.38	0.52	7.55	6.54	7.89	7.22	7.71	
Pz	Study group, eyes open	24	17.13	0.74	17	15.81	18.5	16.75	17.54	p<0.001 *
	Control group	24	6.48	0.87	6.67	5.24	7.79	5.64	7.07	
T3	Study group, eyes open	24	17.95	0.68	17.78	16.77	19	17.62	18.42	p<0.001 *
	Control group	24	7.13	1	6.7	5.89	8.87	6.48	7.79	
T4	Study group, eyes open	24	15.89	3.31	17.61	9.38	18.33	15.37	18.06	p<0.001 *
	Control group	24	7	0.48	7.11	6.02	7.66	6.77	7.33	
O1	Study group, eyes open	24	17.32	0.88	17.41	15.84	18.55	16.75	17.97	p<0.001 *
	Control group	24	6.06	0.85	5.69	5.05	7.86	5.49	6.4	
O2	Study group, eyes open	24	16.81	1	16.92	14.76	18.29	16.26	17.39	p<0.001 *
	Control group	24	6.06	0.83	5.75	5.06	7.75	5.5	6.51	

p - Mann-Whitney test, SD - standard deviation, Q1 - lower quartile, Q3 - upper quartile.

*statistically significant (p<0.05).Values represent the wave amplitude (in µV) with key distribution parameters (including median and quartiles).

### 3.3 Alpha – eyes-open

**Figure 3:**
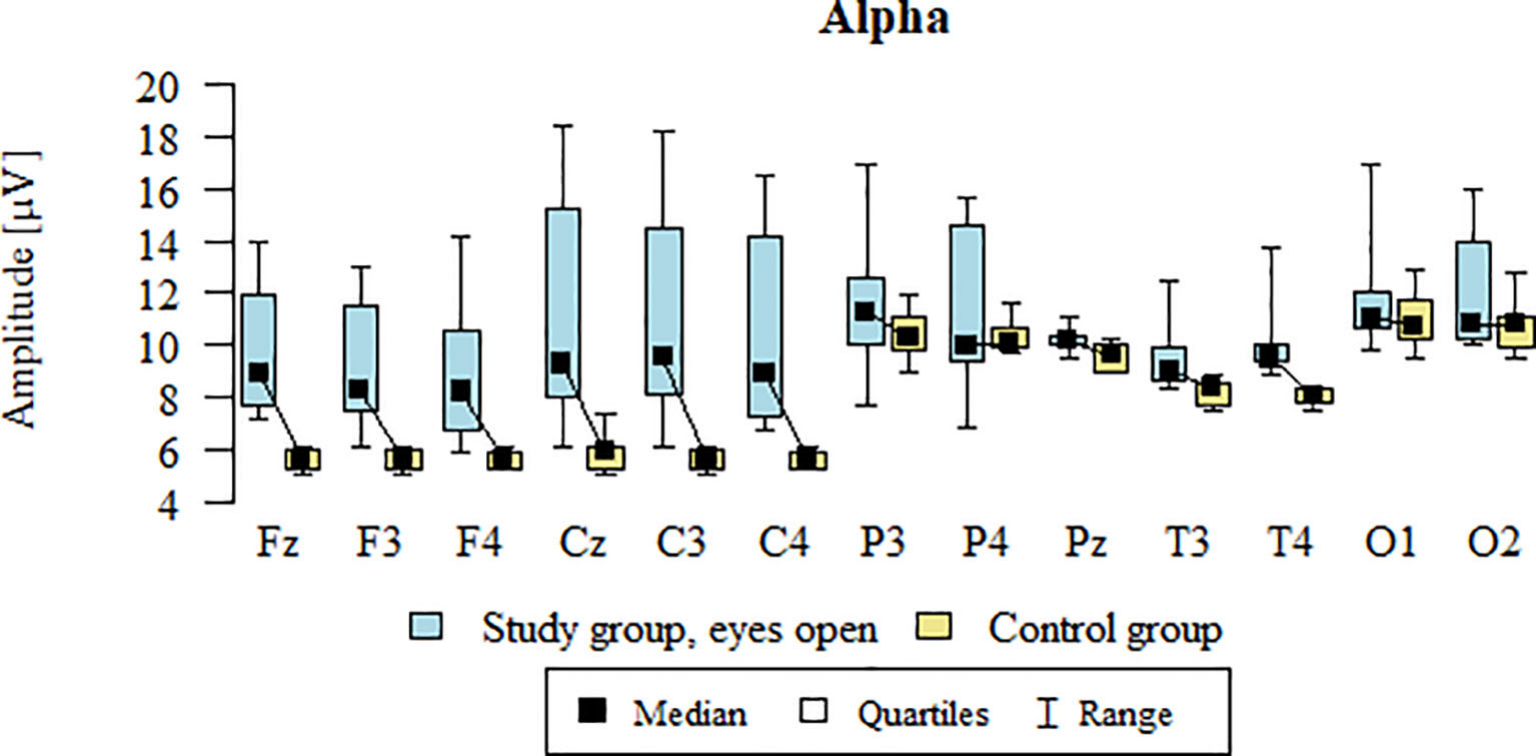
Alpha wave amplitude (µV) in the eyes-open condition for the ASD group (blue) and control group (yellow).

Values of p < 0.05 indicate statistically significant differences. In the eyes-open condition, Alpha wave amplitude was significantly higher in all frontal (Fz, F3, F4), central (Cz, C3, C4), parietal (P3, Pz, P4), temporal (T3, T4), and occipital (O1, O2) sites in the ASD group compared to controls (**Table 6**; **Figure 3**).

**Table 6:** Results of Alpha wave study (waves from channels Fz, F3, F4, Cz, C3, C4, P3, Pz, P4, T3, T4, O1, O2) with eyes open.

Parameter	Group	N	Mean	SD	Median	Min	Max	Q1	Q3	p
Fz	Study group, eyes open	24	9.8	2.45	8.93	7.13	13.96	7.7	11.94	p<0.001 *
	Control group	24	5.63	0.44	5.68	5.02	6.13	5.33	5.98	
F3	Study group, eyes open	24	9.24	2.29	8.29	6.13	13.03	7.49	11.54	p<0.001 *
	Control group	24	5.63	0.44	5.68	5.02	6.13	5.33	5.98	
F4	Study group, eyes open	24	8.84	2.56	8.25	5.95	14.17	6.79	10.59	p<0.001 *
	Control group	24	5.66	0.37	5.58	5.29	6.17	5.33	5.92	
Cz	Study group, eyes open	24	11.2	4.21	9.31	6.13	18.45	8.06	15.26	p<0.001 *
	Control group	24	5.79	0.67	5.93	5.02	7.43	5.33	6.13	
C3	Study group, eyes open	24	11.11	3.99	9.54	6.13	18.15	8.09	14.49	p<0.001 *
	Control group	24	5.63	0.44	5.68	5.02	6.13	5.33	5.98	
C4	Study group, eyes open	24	10.4	3.68	8.95	6.73	16.55	7.26	14.21	p<0.001 *
	Control group	24	5.66	0.37	5.58	5.29	6.17	5.33	5.92	
P3	Study group, eyes open	24	11.56	2.45	11.28	7.72	16.88	10.09	12.61	p=0.048 *
	Control group	24	10.38	0.93	10.28	9.02	11.93	9.8	11.13	
P4	Study group, eyes open	24	11.17	3	10	6.9	15.64	9.38	14.6	p=0.91
	Control group	24	10.34	0.57	10.09	9.72	11.59	9.92	10.63	
Pz	Study group, eyes open	24	10.23	0.45	10.17	9.48	11.14	10.01	10.33	p<0.001 *
	Control group	24	9.58	0.51	9.63	8.98	10.24	9.04	10.07	
T3	Study group, eyes open	24	9.6	1.34	9.07	8.34	12.49	8.63	9.97	p<0.001 *
	Control group	24	8.24	0.47	8.39	7.5	8.92	7.72	8.54	
T4	Study group, eyes open	24	10.12	1.41	9.51	8.89	13.79	9.38	10.07	p<0.001 *
	Control group	24	8.06	0.29	8.09	7.52	8.48	7.83	8.3	
O1	Study group, eyes open	24	11.93	2.25	11.06	9.84	16.9	10.65	12.08	p=0.288
	Control group	24	10.97	0.98	10.74	9.53	12.88	10.27	11.73	
O2	Study group, eyes open	24	11.97	2.19	10.82	10.03	15.95	10.29	13.93	p=0.101
	Control group	24	10.75	0.93	10.82	9.48	12.83	9.91	11.1	

p - Mann-Whitney test, SD - standard deviation, Q1 - lower quartile, Q3 - upper quartile.

*statistically significant (p<0.05). Values represent the wave amplitude (in µV) with key distribution parameters (including median and quartiles).

### 3.4 SMR – eyes-open

**Figure 4:**
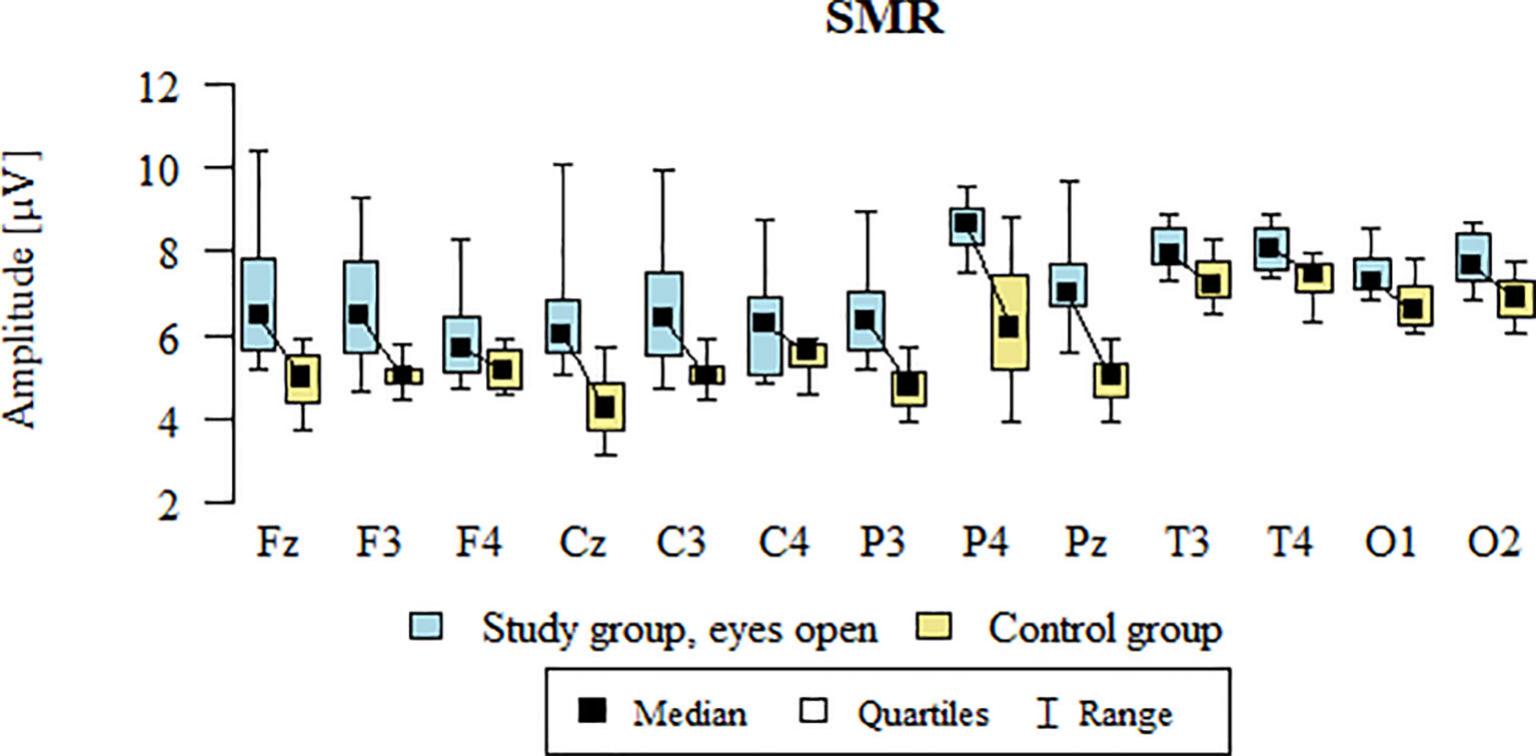
SMR wave amplitude (µV) in the eyes-open condition for the ASD group (blue) and control group (yellow).

Values of p < 0.05 indicate statistically significant differences. In the eyes-open condition, SMR wave amplitude was significantly higher in all frontal (Fz, F3, F4), central (Cz, C3, C4), parietal (P3, Pz, P4), temporal (T3, T4), and occipital (O1, O2) sites in the ASD group compared to controls (**Table 7**; **Figure 4**).

**Table 7:** Results of the SMR wave study (waves from channels Fz, F3, F4, Cz, C3, C4, P3, Pz, P4, T3, T4, O1, O2) with eyes open.

Parameter	Group	N	Mean	SD	Median	Min	Max	Q1	Q3	p
Fz	Study group, eyes open	24	6.95	1.7	6.47	5.19	10.39	5.63	7.81	p<0.001 *
	Control group	24	4.9	0.84	4.97	3.73	5.92	4.37	5.51	
F3	Study group, eyes open	24	6.71	1.41	6.46	4.63	9.31	5.59	7.76	p<0.001 *
	Control group	24	5.04	0.48	5	4.44	5.75	4.85	5.2	
F4	Study group, eyes open	24	5.91	1.09	5.68	4.72	8.28	5.1	6.42	p=0.013 *
	Control group	24	5.2	0.58	5.16	4.58	5.92	4.69	5.67	
Cz	Study group, eyes open	24	6.47	1.43	6.03	5.07	10.11	5.54	6.85	p<0.001 *
	Control group	24	4.34	0.98	4.25	3.12	5.73	3.72	4.87	
C3	Study group, eyes open	24	6.76	1.76	6.43	4.71	9.95	5.5	7.49	p<0.001 *
	Control group	24	5.09	0.55	5	4.44	5.93	4.85	5.24	
C4	Study group, eyes open	24	6.28	1.36	6.26	4.83	8.76	5.02	6.92	p=0.026 *
	Control group	24	5.42	0.52	5.6	4.58	5.92	5.25	5.78	
P3	Study group, eyes open	24	6.58	1.1	6.35	5.17	8.97	5.67	7.03	p<0.001 *
	Control group	24	4.74	0.62	4.78	3.92	5.73	4.31	5.1	
P4	Study group, eyes open	24	8.58	0.63	8.64	7.5	9.54	8.13	9.05	p<0.001 *
	Control group	24	6.21	1.51	6.17	3.92	8.83	5.17	7.46	
Pz	Study group, eyes open	24	7.36	1.23	6.98	5.61	9.68	6.68	7.66	p<0.001 *
	Control group	24	4.94	0.6	5	3.93	5.92	4.54	5.29	
T3	Study group, eyes open	24	8.1	0.51	7.93	7.32	8.91	7.73	8.56	p<0.001 *
	Control group	24	7.28	0.53	7.19	6.5	8.26	6.91	7.78	
T4	Study group, eyes open	24	8.07	0.53	8.08	7.35	8.89	7.57	8.52	p<0.001 *
	Control group	24	7.32	0.5	7.44	6.33	7.96	7.04	7.68	
O1	Study group, eyes open	24	7.48	0.54	7.27	6.84	8.54	7.09	7.85	p<0.001 *
	Control group	24	6.74	0.6	6.59	6.06	7.86	6.23	7.18	
O2	Study group, eyes open	24	7.81	0.63	7.66	6.83	8.67	7.31	8.45	p<0.001 *
	Control group	24	6.85	0.58	6.9	6.01	7.76	6.44	7.3	

p - Mann-Whitney test, SD - standard deviation, Q1 - lower quartile, Q3 - upper quartile.

*statistically significant (p<0.05).The values express the amplitude of the wave (in µV) with the main distribution parameters (including median and quartiles).

### 3.5 Beta1 – eyes-open

**Figure 5:**
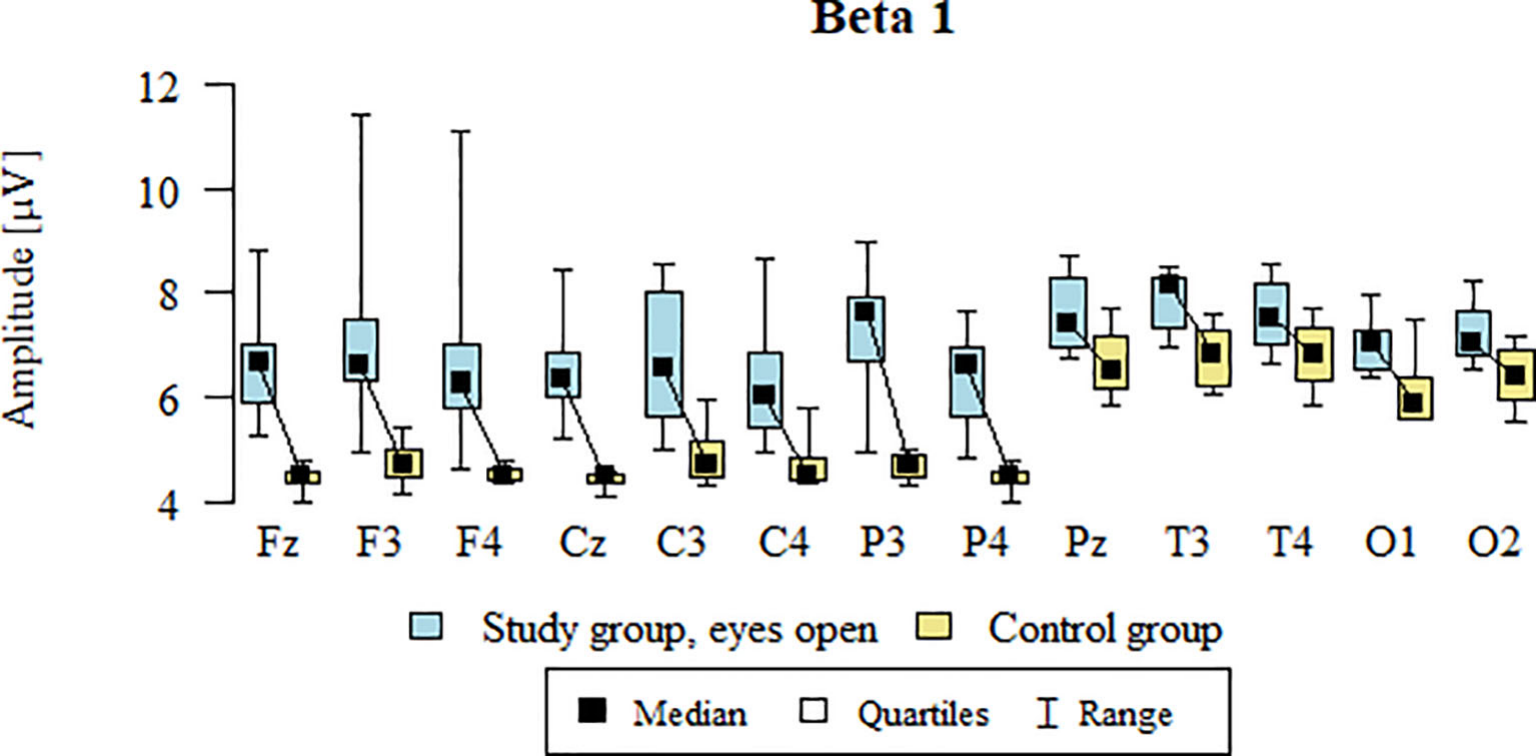
Beta1 wave amplitude (µV) in the eyes-open condition for the ASD group (blue) and control group (yellow).

Values of p < 0.05 indicate statistically significant differences. In the eyes-open condition, Beta1 wave amplitude was significantly higher in all frontal (Fz, F3, F4), central (Cz, C3, C4), parietal (P3, Pz, P4), temporal (T3, T4), and occipital (O1, O2) sites in the ASD group compared to controls (**Table 8**; **Figure 5**).

**Table 8:** Results of the Beta1 wave study (waves from channels Fz, F3, F4, Cz, C3, C4, P3, Pz, P4, T3, T4, O1, O2) with eyes open.

Parameter	Group	N	Mean	SD	Median	Min	Max	Q1	Q3	p
Fz	Study group, eyes open	24	6.62	0.98	6.66	5.29	8.8	5.92	7.04	p<0.001 *
	Control group	24	4.45	0.3	4.48	4.01	4.82	4.35	4.59	
F3	Study group, eyes open	24	7.09	1.59	6.61	4.98	11.41	6.35	7.5	p<0.001 *
	Control group	24	4.76	0.46	4.72	4.18	5.41	4.46	5.02	
F4	Study group, eyes open	24	6.81	1.75	6.28	4.63	11.1	5.8	7.03	p<0.001 *
	Control group	24	4.56	0.17	4.53	4.39	4.82	4.44	4.65	
Cz	Study group, eyes open	24	6.46	0.91	6.34	5.21	8.45	6.04	6.87	p<0.001 *
	Control group	24	4.41	0.18	4.48	4.12	4.56	4.38	4.52	
C3	Study group, eyes open	24	6.74	1.29	6.58	5.01	8.55	5.66	8.03	p<0.001 *
	Control group	24	4.94	0.65	4.72	4.32	5.98	4.5	5.16	
C4	Study group, eyes open	24	6.25	1.11	6.04	4.93	8.64	5.42	6.88	p<0.001 *
	Control group	24	4.79	0.61	4.48	4.36	5.82	4.43	4.84	
P3	Study group, eyes open	24	7.29	1.09	7.64	4.98	9	6.69	7.91	p<0.001 *
	Control group	24	4.7	0.28	4.72	4.32	5.01	4.5	4.92	
P4	Study group, eyes open	24	6.4	0.85	6.62	4.87	7.66	5.62	6.98	p<0.001 *
	Control group	24	4.45	0.3	4.48	4.01	4.82	4.35	4.59	
Pz	Study group, eyes open	24	7.54	0.72	7.4	6.73	8.71	6.97	8.3	p=0.001 *
	Control group	24	6.68	0.61	6.52	5.88	7.7	6.16	7.18	
T3	Study group, eyes open	24	7.84	0.56	8.17	6.97	8.49	7.34	8.3	p<0.001 *
	Control group	24	6.8	0.56	6.85	6.08	7.59	6.22	7.28	
T4	Study group, eyes open	24	7.57	0.63	7.54	6.64	8.53	7.04	8.21	p=0.001 *
	Control group	24	6.86	0.61	6.85	5.87	7.72	6.32	7.34	
O1	Study group, eyes open	24	7.06	0.53	7.05	6.37	7.98	6.56	7.28	p<0.001 *
	Control group	24	6.15	0.66	5.89	5.58	7.48	5.61	6.38	
O2	Study group, eyes open	24	7.23	0.59	7.04	6.52	8.24	6.82	7.66	p<0.001 *
	Control group	24	6.41	0.57	6.42	5.53	7.18	5.98	6.93	

p - Mann-Whitney test, SD - standard deviation, Q1 - lower quartile, Q3 - upper quartile.

*statistically significant (p<0.05).The values express the amplitude of the wave (in µV) with the main distribution parameters (including median and quartiles).

### 3.6 Beta2 – eyes-open

**Figure 6:**
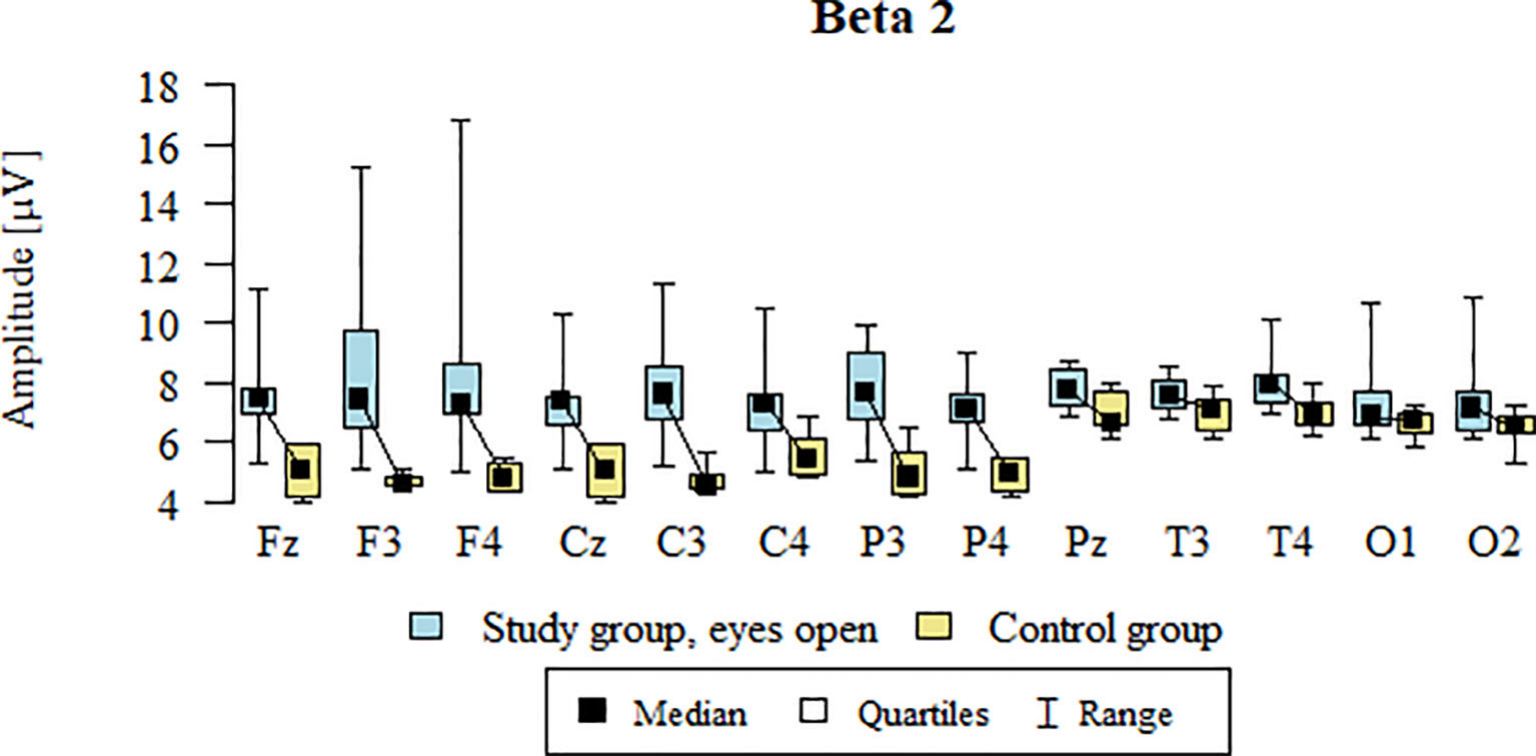
Beta2 wave amplitude (µV) in the eyes-open condition for the ASD group (blue) and control group (yellow).

Values of p < 0.05 indicate statistically significant differences. In the eyes-open condition, Beta2 wave amplitude was significantly higher in all frontal (Fz, F3, F4), central (Cz, C3, C4), parietal (P3, Pz, P4), temporal (T3, T4), and occipital (O1, O2) sites in the ASD group compared to controls (**Table 9**; **Figure 6**).

**Table 9:** Results of the Beta2 wave study (waves from channels Fz, F3, F4, Cz, C3, C4, P3, Pz, P4, T3, T4, O1, O2) with eyes open.

Parameter	Group	N	Mean	SD	Median	Min	Max	Q1	Q3	p
Fz	Study group, eyes open	24	7.66	1.58	7.46	5.33	11.11	6.92	7.82	p<0.001 *
	Control group	24	5.02	0.93	5.05	4.01	5.96	4.16	5.91	
F3	Study group, eyes open	24	8.69	3.27	7.44	5.09	15.19	6.51	9.77	p<0.001 *
	Control group	24	4.72	0.25	4.62	4.51	5.12	4.55	4.8	
F4	Study group, eyes open	24	8.78	3.64	7.31	4.99	16.75	6.99	8.63	p<0.001 *
	Control group	24	4.87	0.51	4.81	4.34	5.51	4.4	5.28	
Cz	Study group, eyes open	24	7.3	1.36	7.38	5.16	10.31	6.58	7.5	p<0.001 *
	Control group	24	5.02	0.93	5.05	4.01	5.96	4.16	5.91	
C3	Study group, eyes open	24	7.67	1.66	7.62	5.23	11.29	6.77	8.54	p<0.001 *
	Control group	24	4.8	0.54	4.59	4.32	5.69	4.46	4.92	
C4	Study group, eyes open	24	7.18	1.4	7.33	5.03	10.52	6.45	7.62	p<0.001 *
	Control group	24	5.65	0.85	5.43	4.82	6.91	4.93	6.14	
P3	Study group, eyes open	24	7.82	1.42	7.63	5.4	9.96	6.83	9.03	p<0.001 *
	Control group	24	5.09	0.96	4.84	4.17	6.51	4.28	5.64	
P4	Study group, eyes open	24	7.12	1.07	7.14	5.11	8.98	6.66	7.58	p<0.001 *
	Control group	24	4.92	0.59	4.97	4.22	5.51	4.41	5.49	
Pz	Study group, eyes open	24	7.8	0.67	7.73	6.89	8.75	7.26	8.43	p<0.001 *
	Control group	24	6.97	0.68	6.66	6.1	7.98	6.57	7.71	
T3	Study group, eyes open	24	7.62	0.57	7.54	6.83	8.53	7.13	8.09	p=0.003 *
	Control group	24	7.01	0.61	7.14	6.14	7.87	6.43	7.44	
T4	Study group, eyes open	24	7.95	0.84	7.96	6.95	10.14	7.34	8.27	p<0.001 *
	Control group	24	7.01	0.55	6.97	6.21	7.96	6.64	7.32	
O1	Study group, eyes open	24	7.46	1.49	6.89	6.09	10.65	6.64	7.72	p=0.049 *
	Control group	24	6.66	0.49	6.72	5.85	7.29	6.3	6.94	
O2	Study group, eyes open	24	7.34	1.28	7.16	6.09	10.89	6.43	7.72	p=0.014 *
	Control group	24	6.5	0.54	6.54	5.34	7.26	6.33	6.85	

p - Mann-Whitney test, SD - standard deviation, Q1 - lower quartile, Q3 - upper quartile.

*statistically significant (p<0.05). The values express the amplitude of the wave (in µV) with the main distribution parameters (including median and quartiles).

### 3.7 Delta – eyes-closed

**Figure 7:**
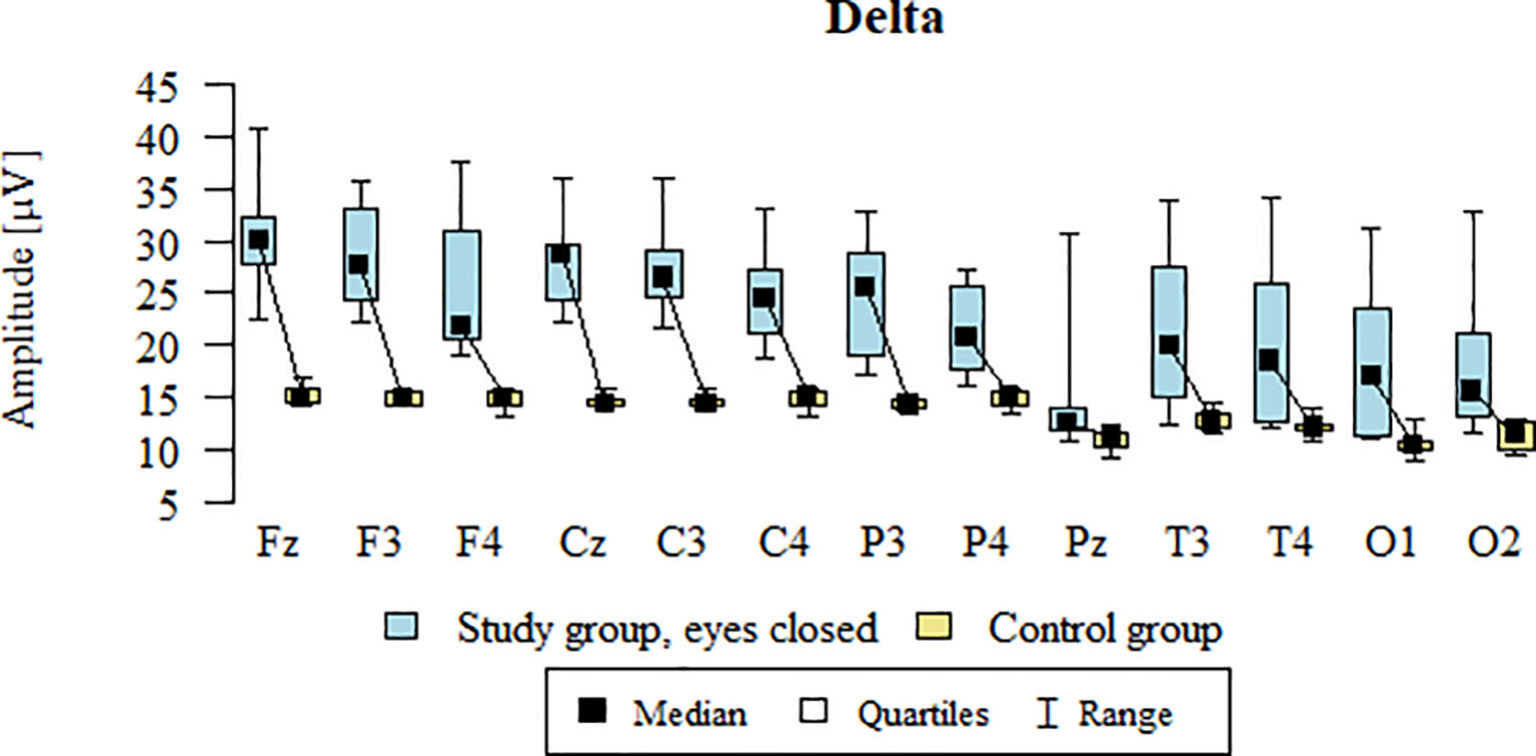
Delta wave amplitude (µV) in the eyes-closed condition for the ASD group (blue) and control group (yellow).

Values of p < 0.05 indicate statistically significant differences: the amplitude of the Delta wave was significantly higher in all frontal (Fz, F3, F4), central (Cz, C3, C4), parietal (P3, Pz, P4), temporal (T3, T4), and occipital (O1, O2) leads in the ASD group compared to the control group with eyes closed (**Table 10**; **Figure 7**).

**Table 10:** Results of the Delta wave study (waves from channels Fz, F3, F4, Cz, C3, C4, P3, Pz, P4, T3, T4, O1, O2) with eyes closed.

Parameter	Group	N	Mean	SD	Median	Min	Max	Q1	Q3	p
Fz	Study group, eyes closed	24	30.2	4.62	30.1	22.47	40.66	27.89	32.38	p<0.001 *
	Control group	24	15.3	1.09	15	14.23	16.98	14.48	15.82	
F3	Study group, eyes closed	24	28.37	4.85	27.6	22.21	35.62	24.33	33.15	p<0.001 *
	Control group	24	15.05	0.75	15	14.23	15.98	14.38	15.66	
F4	Study group, eyes closed	24	25.7	7.03	21.82	19.08	37.45	20.56	31	p<0.001 *
	Control group	24	14.82	1.03	15.01	13.28	15.98	14.29	15.54	
Cz	Study group, eyes closed	24	28.19	4.09	28.72	22.3	35.99	24.26	29.68	p<0.001 *
	Control group	24	14.8	0.71	14.5	14.23	15.98	14.38	14.91	
C3	Study group, eyes closed	24	27.29	4.31	26.44	21.74	36.05	24.59	29.15	p<0.001 *
	Control group	24	14.8	0.71	14.5	14.23	15.98	14.38	14.91	
C4	Study group, eyes closed	24	24.73	4.39	24.55	18.8	33.14	21.08	27.32	p<0.001 *
	Control group	24	14.87	1.06	15.11	13.28	15.98	14.29	15.69	
P3	Study group, eyes closed	24	24.13	5.49	25.45	17.24	32.69	18.92	28.91	p<0.001 *
	Control group	24	14.43	0.7	14.5	13.43	15.29	14.03	14.89	
P4	Study group, eyes closed	24	21.38	4.37	20.87	16.03	27.2	17.72	25.56	p<0.001 *
	Control group	24	14.89	0.97	15.05	13.48	15.98	14.34	15.6	
Pz	Study group, eyes closed	24	14.15	5.25	12.46	10.71	30.74	11.93	14.09	p<0.001 *
	Control group	24	11.04	0.87	11.36	9.33	12.11	10.41	11.64	
T3	Study group, eyes closed	24	21.26	7.28	20.07	12.36	33.99	15.13	27.47	p<0.001 *
	Control group	24	12.88	0.92	12.71	11.5	14.62	12.18	13.5	
T4	Study group, eyes closed	24	20.1	7.63	18.49	12.18	34.05	12.82	25.81	p<0.001 *
	Control group	24	12.29	0.95	12.15	10.77	14.11	11.84	12.41	
O1	Study group, eyes closed	24	18.29	7.1	17.09	11.19	31.14	11.43	23.62	p<0.001 *
	Control group	24	10.58	1.13	10.37	9.04	12.83	9.94	10.95	
O2	Study group, eyes closed	24	18.51	7.13	15.61	11.5	32.69	13.15	21.26	p<0.001 *
	Control group	24	11.33	1.32	11.46	9.59	12.93	9.95	12.73	

p - Mann-Whitney test, SD - standard deviation, Q1 - lower quartile, Q3 - upper quartile.

*statistically significant (p<0.05). The values express the amplitude of the wave (in µV) with the main distribution parameters (including median and quartiles).

### 3.8 Theta – eyes-closed

**Figure 8:**
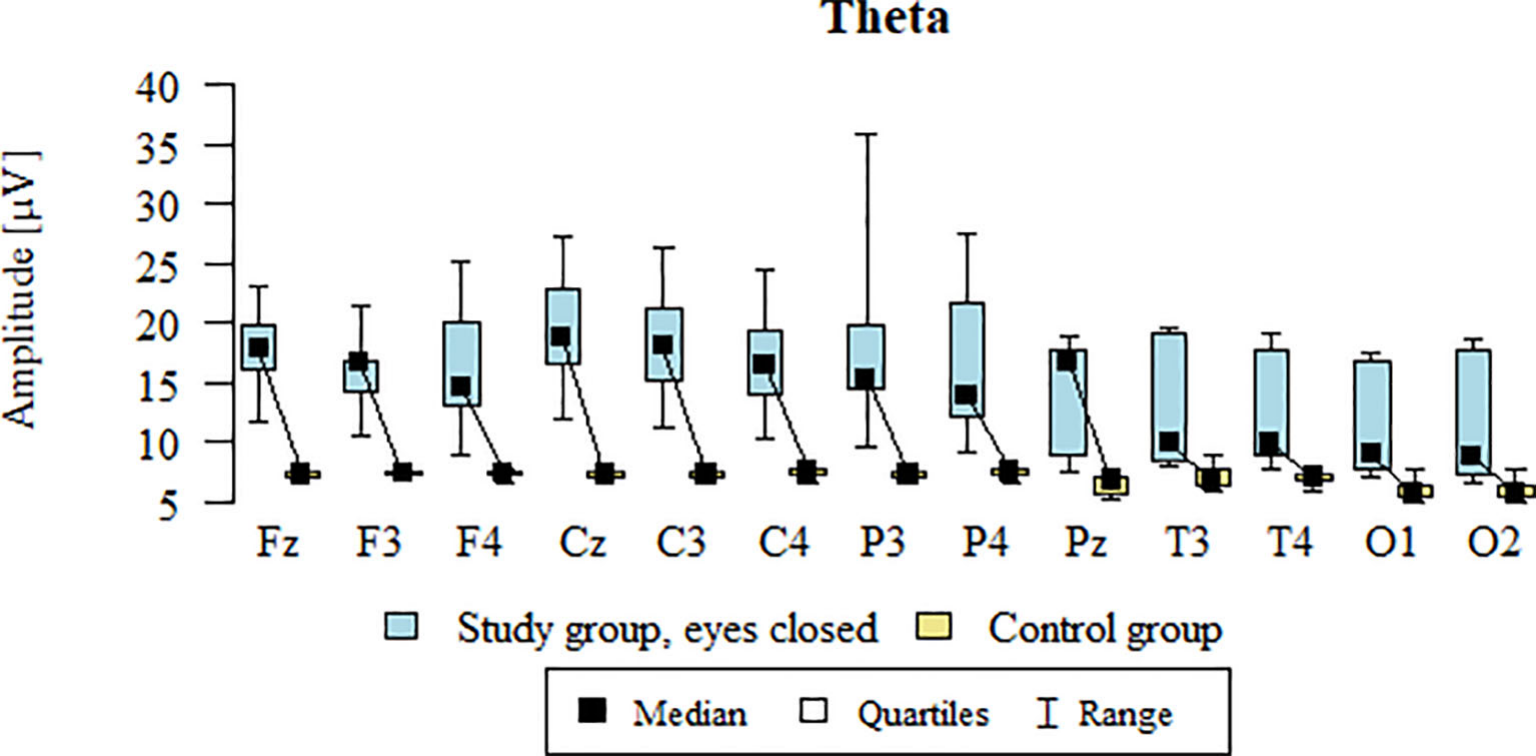
Theta wave amplitude (µV) in the eyes-closed condition for the ASD group (blue) and control group (yellow).

Values of p < 0.05 indicate statistically significant differences: the amplitude of the Theta wave was significantly higher in all frontal (Fz, F3, F4), central (Cz, C3, C4), parietal (P3, Pz, P4), temporal (T3, T4), and occipital (O1, O2) leads in the ASD group compared to the control group with eyes closed (**Table 11**; **Figure 8**).

**Table 11:** Results of the Theta wave study (waves from channels Fz, F3, F4, Cz, C3, C4, P3, Pz, P4, T3, T4, O1, O2) with eyes closed.

Parameter	Group	N	Mean	SD	Median	Min	Max	Q1	Q3	p
Fz	Study group, eyes closed	24	17.7	3.29	17.84	11.63	23.06	16.12	19.86	p<0.001 *
	Control group	24	7.36	0.16	7.35	7.18	7.54	7.2	7.5	
F3	Study group, eyes closed	24	16.1	2.96	16.66	10.56	21.52	14.17	16.9	p<0.001 *
	Control group	24	7.41	0.13	7.44	7.21	7.54	7.34	7.5	
F4	Study group, eyes closed	24	15.99	4.85	14.7	9.04	25.25	13.23	20.06	p<0.001 *
	Control group	24	7.34	0.5	7.46	6.54	7.89	7.22	7.58	
Cz	Study group, eyes closed	24	19.42	4.48	18.78	11.97	27.34	16.6	22.93	p<0.001 *
	Control group	24	7.33	0.2	7.35	7.08	7.54	7.18	7.5	
C3	Study group, eyes closed	24	18.29	4.47	18.05	11.29	26.25	15.3	21.27	p<0.001 *
	Control group	24	7.33	0.2	7.35	7.08	7.54	7.18	7.5	
C4	Study group, eyes closed	24	16.69	4.15	16.51	10.33	24.53	14.1	19.46	p<0.001 *
	Control group	24	7.38	0.52	7.55	6.54	7.89	7.22	7.71	
P3	Study group, eyes closed	24	18.02	6.78	15.32	9.68	35.84	14.61	19.76	p<0.001 *
	Control group	24	7.33	0.2	7.35	7.08	7.54	7.18	7.5	
P4	Study group, eyes closed	24	16.36	5.7	13.97	9.11	27.53	12.11	21.62	p<0.001 *
	Control group	24	7.38	0.52	7.55	6.54	7.89	7.22	7.71	
Pz	Study group, eyes closed	24	13.87	4.75	16.84	7.46	18.83	9.02	17.75	p<0.001 *
	Control group	24	6.48	0.87	6.67	5.24	7.79	5.64	7.07	
T3	Study group, eyes closed	24	13.2	5.26	10.02	8.03	19.64	8.47	19.09	p<0.001 *
	Control group	24	7.13	1	6.7	5.89	8.87	6.48	7.79	
T4	Study group, eyes closed	24	12.22	4.39	9.87	7.75	19.23	9.05	17.8	p<0.001 *
	Control group	24	7	0.48	7.11	6.02	7.66	6.77	7.33	
O1	Study group, eyes closed	24	11.2	4.36	9.05	6.99	17.51	7.77	16.78	p<0.001 *
	Control group	24	6.06	0.85	5.69	5.05	7.86	5.49	6.4	
O2	Study group, eyes closed	24	12.07	5.25	8.9	6.71	18.63	7.41	17.85	p<0.001 *
	Control group	24	6.06	0.83	5.75	5.06	7.75	5.5	6.51	

p - Mann-Whitney test, SD - standard deviation, Q1 - lower quartile, Q3 - upper quartile.

*statistically significant (p<0.05).The values express the amplitude of the wave (in µV) with the main distribution parameters (including median and quartiles).

### 3.9 Alpha – eyes-closed

**Figure 9:**
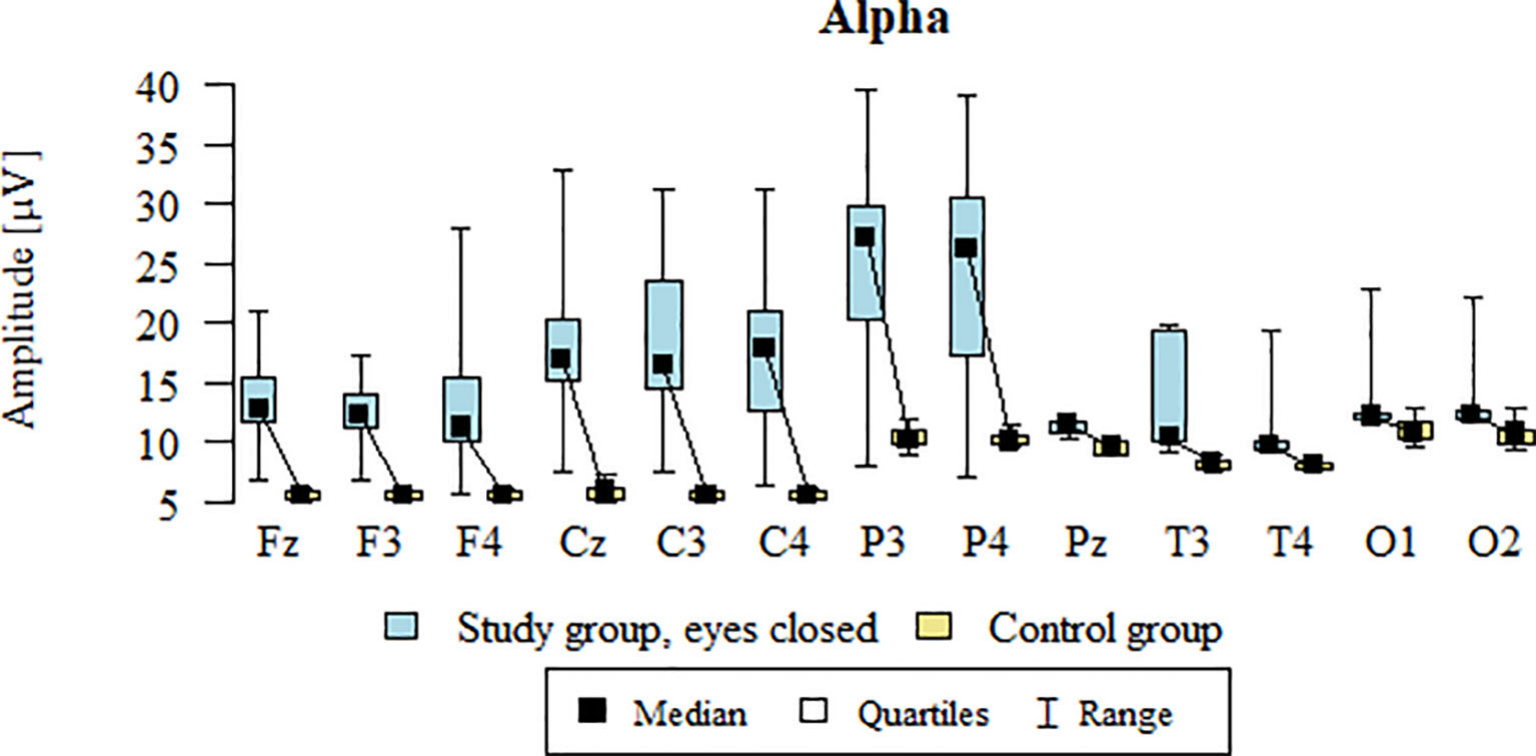
Alpha wave amplitude (µV) in the eyes-closed condition for the ASD group (blue) and control group (yellow).

Values of p < 0.05 indicate statistically significant differences: the amplitude of the Alpha wave was significantly higher in all frontal (Fz, F3, F4), central (Cz, C3, C4), parietal (P3, Pz, P4), temporal (T3, T4), and occipital (O1, O2) leads in the ASD group compared to the control group with eyes closed - **Table 12**; **Figure 9**.

**Table 12:** Results of the Alpha wave study (waves from channels Fz, F3, F4, Cz, C3, C4, P3, Pz, P4, T3, T4, O1, O2) with eyes closed.

Parameter	Group	N	Mean	SD	Median	Min	Max	Q1	Q3	p
Fz	Study group, eyes closed	24	13.49	4.18	12.81	6.86	20.88	11.75	15.44	p<0.001 *
	Control group	24	5.63	0.44	5.68	5.02	6.13	5.33	5.98	
F3	Study group, eyes closed	24	12.62	3.19	12.23	6.76	17.34	11.38	14.04	p<0.001 *
	Control group	24	5.63	0.44	5.68	5.02	6.13	5.33	5.98	
F4	Study group, eyes closed	24	13.02	5.74	11.27	5.68	27.83	10.22	15.45	p<0.001 *
	Control group	24	5.66	0.37	5.58	5.29	6.17	5.33	5.92	
Cz	Study group, eyes closed	24	18.38	6.46	16.94	7.63	32.83	15.16	20.34	p<0.001 *
	Control group	24	5.79	0.67	5.93	5.02	7.43	5.33	6.13	
C3	Study group, eyes closed	24	18.61	7.68	16.57	7.63	31.28	14.58	23.51	p<0.001 *
	Control group	24	5.63	0.44	5.68	5.02	6.13	5.33	5.98	
C4	Study group, eyes closed	24	17.41	7.53	17.91	6.35	31.11	12.77	20.92	p<0.001 *
	Control group	24	5.66	0.37	5.58	5.29	6.17	5.33	5.92	
P3	Study group, eyes closed	24	25.29	8.73	27.14	7.97	39.56	20.34	29.79	p<0.001 *
	Control group	24	10.38	0.93	10.28	9.02	11.93	9.8	11.13	
P4	Study group, eyes closed	24	23.92	9.63	26.25	7.08	39.05	17.36	30.41	p<0.001 *
	Control group	24	10.34	0.57	10.09	9.72	11.59	9.92	10.63	
Pz	Study group, eyes closed	24	11.23	0.51	11.5	10.31	11.77	10.82	11.63	p<0.001 *
	Control group	24	9.58	0.51	9.63	8.98	10.24	9.04	10.07	
T3	Study group, eyes closed	24	13.23	4.63	10.4	9.15	19.93	10.09	19.27	p<0.001 *
	Control group	24	8.24	0.47	8.39	7.5	8.92	7.72	8.54	
T4	Study group, eyes closed	24	10.53	2.76	9.68	9.22	19.39	9.36	10.14	p<0.001 *
	Control group	24	8.06	0.29	8.09	7.52	8.48	7.83	8.3	
O1	Study group, eyes closed	24	13.05	3.01	12.21	11.69	22.76	11.95	12.45	p<0.001 *
	Control group	24	10.97	0.98	10.74	9.53	12.88	10.27	11.73	
O2	Study group, eyes closed	24	13.07	2.8	12.25	11.71	22.07	11.93	12.63	p<0.001 *
	Control group	24	10.75	0.93	10.82	9.48	12.83	9.91	11.1	

p - Mann-Whitney test, SD - standard deviation, Q1 - lower quartile, Q3 - upper quartile.

*statistically significant (p<0.05).The values express the amplitude of the wave (in µV) with the main distribution parameters (including median and quartiles).

### 3.10 SMR – eyes-closed

**Figure 10:**
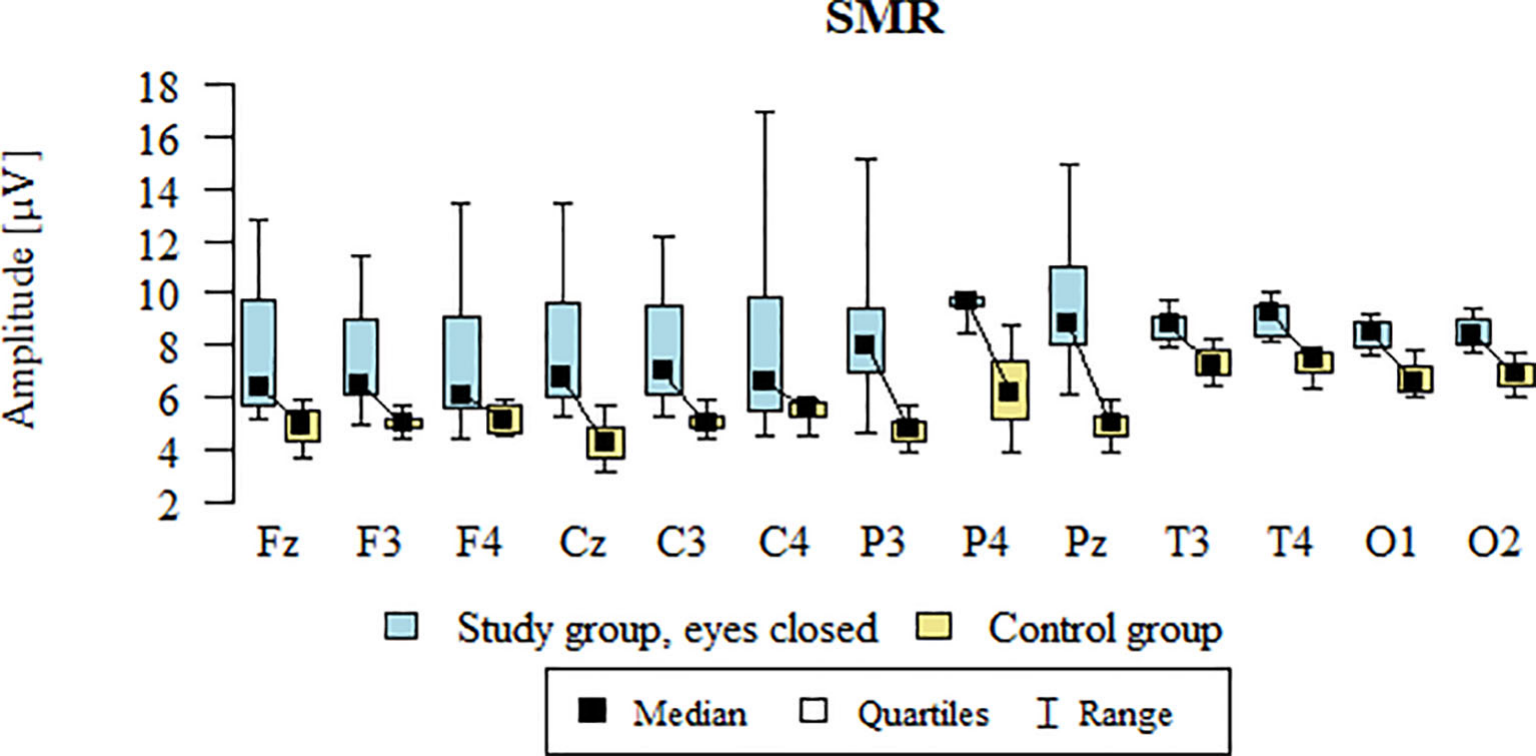
SMR wave amplitude (µV) in the eyes-closed condition for the ASD group (blue) and control group (yellow).

Values of p < 0.05 indicate statistically significant differences: the amplitude of the SMR wave was significantly higher in all frontal (Fz, F3, F4), central (Cz, C3, C4), parietal (P3, Pz, P4), temporal (T3, T4), and occipital (O1, O2) leads in the ASD group compared to the control group with eyes closed - **Table 13**; **Figure 10**.

**Table 13:** Results of the SMR wave study (waves from channels Fz, F3, F4, Cz, C3, C4, P3, Pz, P4, T3, T4, O1, O2) with eyes closed.

Parameter	Group	N	Mean	SD	Median	Min	Max	Q1	Q3	p
Fz	Study group, eyes closed	24	7.66	2.64	6.4	5.17	12.8	5.7	9.75	p<0.001 *
	Control group	24	4.9	0.84	4.97	3.73	5.92	4.37	5.51	
F3	Study group, eyes closed	24	7.49	2.13	6.51	4.95	11.4	6.15	8.95	p<0.001 *
	Control group	24	5.04	0.48	5	4.44	5.75	4.85	5.2	
F4	Study group, eyes closed	24	7.14	2.58	6.09	4.39	13.45	5.61	9.09	p=0.003 *
	Control group	24	5.2	0.58	5.16	4.58	5.92	4.69	5.67	
Cz	Study group, eyes closed	24	7.93	2.6	6.78	5.3	13.39	6.03	9.62	p<0.001 *
	Control group	24	4.34	0.98	4.25	3.12	5.73	3.72	4.87	
C3	Study group, eyes closed	24	7.91	2.34	7	5.33	12.2	6.11	9.52	p<0.001 *
	Control group	24	5.09	0.55	5	4.44	5.93	4.85	5.24	
C4	Study group, eyes closed	24	7.87	3.47	6.6	4.57	16.93	5.5	9.88	p=0.013 *
	Control group	24	5.42	0.52	5.6	4.58	5.92	5.25	5.78	
P3	Study group, eyes closed	24	8.47	2.61	8.02	4.63	15.13	6.94	9.39	p<0.001 *
	Control group	24	4.74	0.62	4.78	3.92	5.73	4.31	5.1	
P4	Study group, eyes closed	24	9.59	0.48	9.7	8.44	10.08	9.53	9.85	p<0.001 *
	Control group	24	6.21	1.51	6.17	3.92	8.83	5.17	7.46	
Pz	Study group, eyes closed	24	9.72	2.76	8.82	6.12	14.93	8.07	11.03	p<0.001 *
	Control group	24	4.94	0.6	5	3.93	5.92	4.54	5.29	
T3	Study group, eyes closed	24	8.78	0.57	8.84	7.98	9.72	8.27	9.09	p<0.001 *
	Control group	24	7.28	0.53	7.19	6.5	8.26	6.91	7.78	
T4	Study group, eyes closed	24	9.03	0.66	9.25	8.19	10	8.35	9.56	p<0.001 *
	Control group	24	7.32	0.5	7.44	6.33	7.96	7.04	7.68	
O1	Study group, eyes closed	24	8.44	0.55	8.48	7.62	9.23	7.88	8.84	p<0.001 *
	Control group	24	6.74	0.6	6.59	6.06	7.86	6.23	7.18	
O2	Study group, eyes closed	24	8.49	0.59	8.44	7.72	9.37	8.02	9	p<0.001 *
	Control group	24	6.85	0.58	6.9	6.01	7.76	6.44	7.3	

p - Mann-Whitney test, SD - standard deviation, Q1 - lower quartile, Q3 - upper quartile.

*statistically significant (p<0.05).The values express the amplitude of the wave (in µV) with the main distribution parameters (including median and quartiles).

### 3.11 Beta1 – eyes-closed

**Figure 11:**
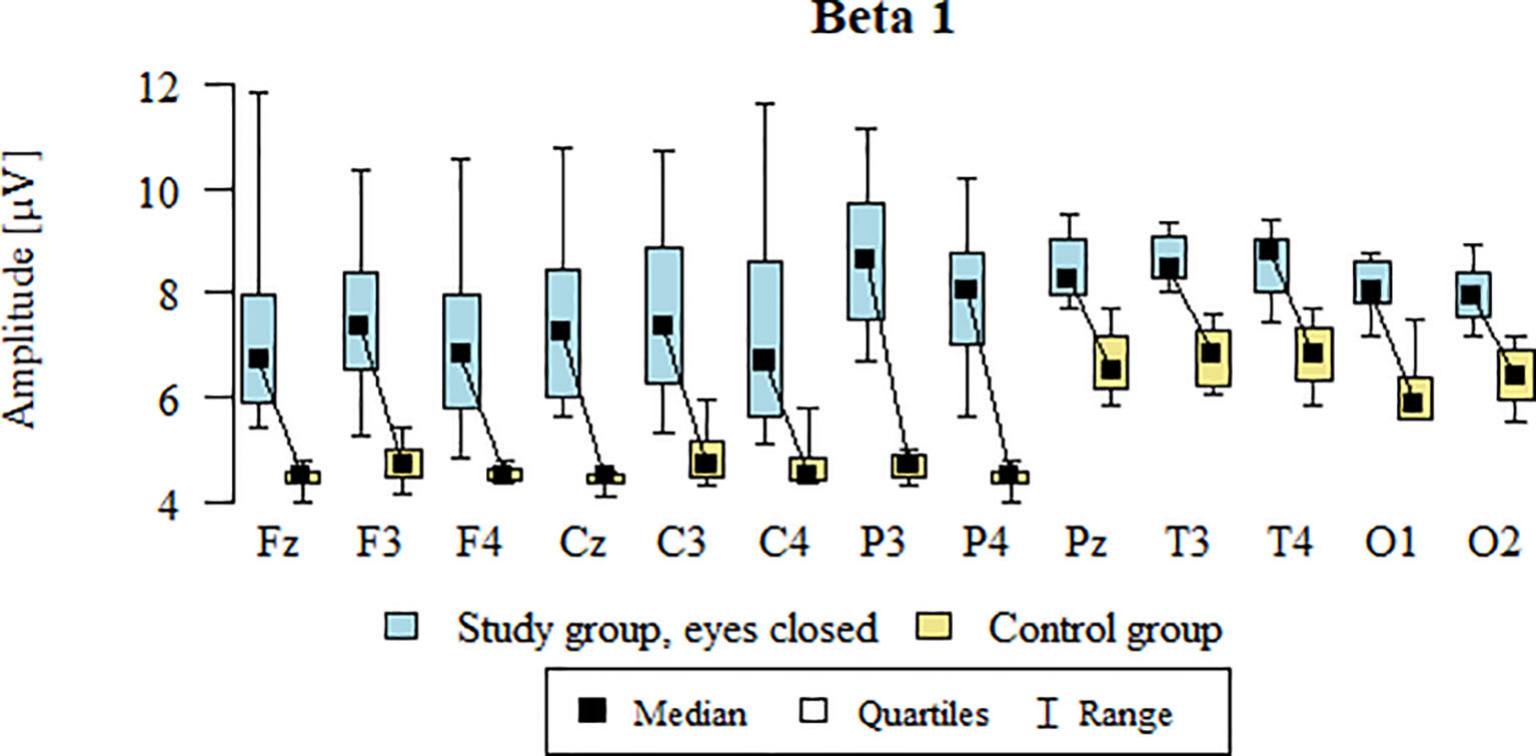
Beta1 wave amplitude (µV) in the eyes-closed condition for the ASD group (blue) and control group (yellow).

Values of p < 0.05 indicate statistically significant differences: the amplitude of the Beta1 wave was significantly higher in all frontal (Fz, F3, F4), central (Cz, C3, C4), parietal (P3, Pz, P4), temporal (T3, T4), and occipital (O1, O2) leads in the ASD group compared to the control group with eyes closed (**Table 14**; **Figure 11**).

**Table 14:** Results of the Beta1 wave study (waves from channels Fz, F3, F4, Cz, C3, C4, P3, Pz, P4, T3, T4, O1, O2) with eyes closed.

Parameter	Group	N	Mean	SD	Median	Min	Max	Q1	Q3	p
Fz	Study group, eyes closed	24	7.34	1.91	6.72	5.43	11.85	5.93	7.95	p<0.001 *
	Control group	24	4.45	0.3	4.48	4.01	4.82	4.35	4.59	
F3	Study group, eyes closed	24	7.57	1.68	7.35	5.28	10.35	6.54	8.42	p<0.001 *
	Control group	24	4.76	0.46	4.72	4.18	5.41	4.46	5.02	
F4	Study group, eyes closed	24	7.04	1.78	6.84	4.85	10.59	5.8	7.99	p<0.001 *
	Control group	24	4.56	0.17	4.53	4.39	4.82	4.44	4.65	
Cz	Study group, eyes closed	24	7.44	1.64	7.25	5.62	10.78	6.01	8.45	p<0.001 *
	Control group	24	4.41	0.18	4.48	4.12	4.56	4.38	4.52	
C3	Study group, eyes closed	24	7.69	1.63	7.36	5.33	10.71	6.27	8.9	p<0.001 *
	Control group	24	4.94	0.65	4.72	4.32	5.98	4.5	5.16	
C4	Study group, eyes closed	24	7.48	2.22	6.7	5.09	11.61	5.63	8.61	p<0.001 *
	Control group	24	4.79	0.61	4.48	4.36	5.82	4.43	4.84	
P3	Study group, eyes closed	24	8.73	1.43	8.62	6.68	11.17	7.5	9.74	p<0.001 *
	Control group	24	4.7	0.28	4.72	4.32	5.01	4.5	4.92	
P4	Study group, eyes closed	24	7.97	1.39	8.07	5.65	10.18	7.02	8.77	p<0.001 *
	Control group	24	4.45	0.3	4.48	4.01	4.82	4.35	4.59	
Pz	Study group, eyes closed	24	8.48	0.59	8.27	7.69	9.49	7.98	9.05	p<0.001 *
	Control group	24	6.68	0.61	6.52	5.88	7.7	6.16	7.18	
T3	Study group, eyes closed	24	8.6	0.47	8.45	8.04	9.34	8.3	9.07	p<0.001 *
	Control group	24	6.8	0.56	6.85	6.08	7.59	6.22	7.28	
T4	Study group, eyes closed	24	8.5	0.69	8.77	7.43	9.39	8	9.04	p<0.001 *
	Control group	24	6.86	0.61	6.85	5.87	7.72	6.32	7.34	
O1	Study group, eyes closed	24	8.13	0.49	8.03	7.2	8.75	7.83	8.63	p<0.001 *
	Control group	24	6.15	0.66	5.89	5.58	7.48	5.61	6.38	
O2	Study group, eyes closed	24	8.01	0.55	7.94	7.17	8.9	7.55	8.39	p<0.001 *
	Control group	24	6.41	0.57	6.42	5.53	7.18	5.98	6.93	

p - Mann-Whitney test, SD - standard deviation, Q1 - lower quartile, Q3 - upper quartile.

*statistically significant (p<0.05).The values express the amplitude of the wave (in µV) with the main distribution parameters (including median and quartiles).

### 3.12 Beta2 – eyes-closed

**Figure 12:**
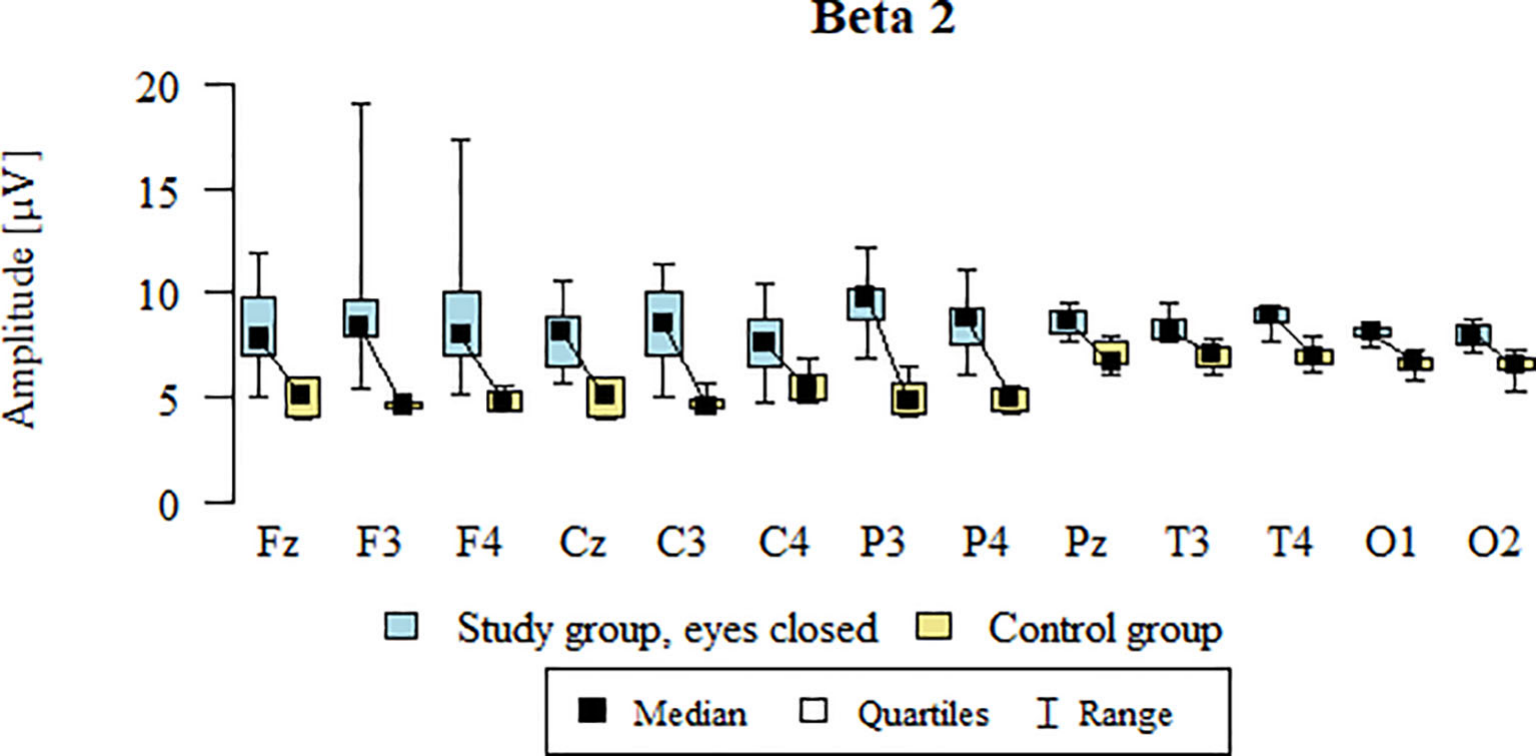
Beta2 wave amplitude (µV) in the eyes-closed condition for the ASD group (blue) and control group (yellow).

Values of p < 0.05 indicate statistically significant differences: the amplitude of the Beta2 wave was significantly higher in all frontal (Fz, F3, F4), central (Cz, C3, C4), parietal (P3, Pz, P4), temporal (T3, T4), and occipital (O1, O2) leads in the ASD group compared to the control group with eyes closed - **Table 15**; **Figure 12**.

**Table 15:** Results of the Beta2 wave study (waves from channels Fz, F3, F4, Cz, C3, C4, P3, Pz, P4, T3, T4, O1, O2) with eyes closed.

Parameter	Group	N	Mean	SD	Median	Min	Max	Q1	Q3	p
Fz	Study group, eyes closed	24	8.37	2.02	7.82	5.08	11.9	7	9.8	p<0.001 *
	Control group	24	5.02	0.93	5.05	4.01	5.96	4.16	5.91	
F3	Study group, eyes closed	24	9.38	3.52	8.37	5.46	19.06	7.9	9.63	p<0.001 *
	Control group	24	4.72	0.25	4.62	4.51	5.12	4.55	4.8	
F4	Study group, eyes closed	24	8.85	3.27	7.98	5.16	17.41	7.08	10.03	p<0.001 *
	Control group	24	4.87	0.51	4.81	4.34	5.51	4.4	5.28	
Cz	Study group, eyes closed	24	8.02	1.63	8.11	5.71	10.6	6.56	8.91	p<0.001 *
	Control group	24	5.02	0.93	5.05	4.01	5.96	4.16	5.91	
C3	Study group, eyes closed	24	8.44	1.98	8.59	5.03	11.4	7.07	10.02	p<0.001 *
	Control group	24	4.8	0.54	4.59	4.32	5.69	4.46	4.92	
C4	Study group, eyes closed	24	7.71	1.69	7.61	4.74	10.51	6.56	8.8	p<0.001 *
	Control group	24	5.65	0.85	5.43	4.82	6.91	4.93	6.14	
P3	Study group, eyes closed	24	9.48	1.57	9.8	6.89	12.24	8.77	10.17	p<0.001 *
	Control group	24	5.09	0.96	4.84	4.17	6.51	4.28	5.64	
P4	Study group, eyes closed	24	8.55	1.5	8.78	6.13	11.16	7.53	9.27	p<0.001 *
	Control group	24	4.92	0.59	4.97	4.22	5.51	4.41	5.49	
Pz	Study group, eyes closed	24	8.62	0.63	8.64	7.74	9.48	8.04	9.2	p<0.001 *
	Control group	24	6.97	0.68	6.66	6.1	7.98	6.57	7.71	
T3	Study group, eyes closed	24	8.36	0.66	8.16	7.68	9.59	7.77	8.75	p<0.001 *
	Control group	24	7.01	0.61	7.14	6.14	7.87	6.43	7.44	
T4	Study group, eyes closed	24	8.82	0.53	8.97	7.72	9.4	8.63	9.23	p<0.001 *
	Control group	24	7.01	0.55	6.97	6.21	7.96	6.64	7.32	
O1	Study group, eyes closed	24	8.14	0.35	8.12	7.45	8.67	7.9	8.4	p<0.001 *
	Control group	24	6.66	0.49	6.72	5.85	7.29	6.3	6.94	
O2	Study group, eyes closed	24	8.01	0.55	8.06	7.09	8.71	7.62	8.49	p<0.001 *
	Control group	24	6.5	0.54	6.54	5.34	7.26	6.33	6.85	

p - Mann-Whitney test, SD - standard deviation, Q1 - lower quartile, Q3 - upper quartile.

*statistically significant (p<0.05). The values express the amplitude of the wave (in µV) with the main distribution parameters (including median and quartiles).

**Figure 13:**
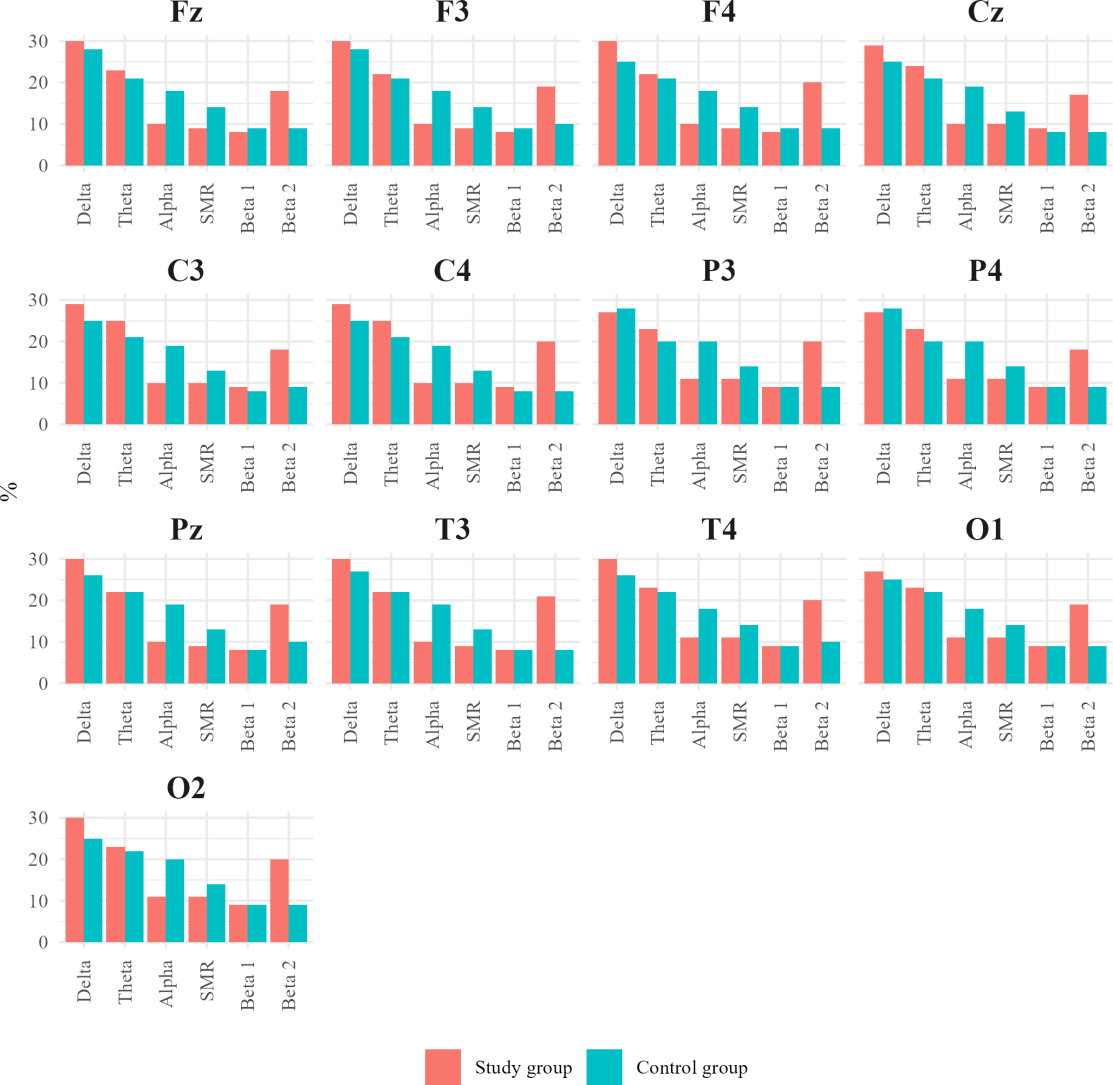
Charts juxtaposing the percentage contribution of Alpha, SMR, and Beta 2 waves in the individual central (Cz, C3, C4), frontal (Fz, F3, F4), parietal (P3, Pz, P4), temporal (T3, T4), and occipital (O1, O2) leads for the ASD group and healthy individuals.

To visually represent the differences in the percentage contribution of brainwaves between the ASD group and the control group, **Figure 13** was created. This figure compares the values obtained in our study for children with ASD with the corresponding values for healthy children.

### 3.13 Percentage of the wave distribution

In analyzing our results, we discovered an interesting trend in the percentage contribution of brain waves. In children with autism spectrum disorder, the main fluctuations in brain wave percentage were observed in the Alpha and Beta2 wave ranges. For Alpha waves, in children with autism or ASD, the percentage contribution decreased to around 10%. Changes in Beta2 waves included an increase in their percentage to about 15–20% compared to the norm, according to Sterman (14).

**Figure 14:**
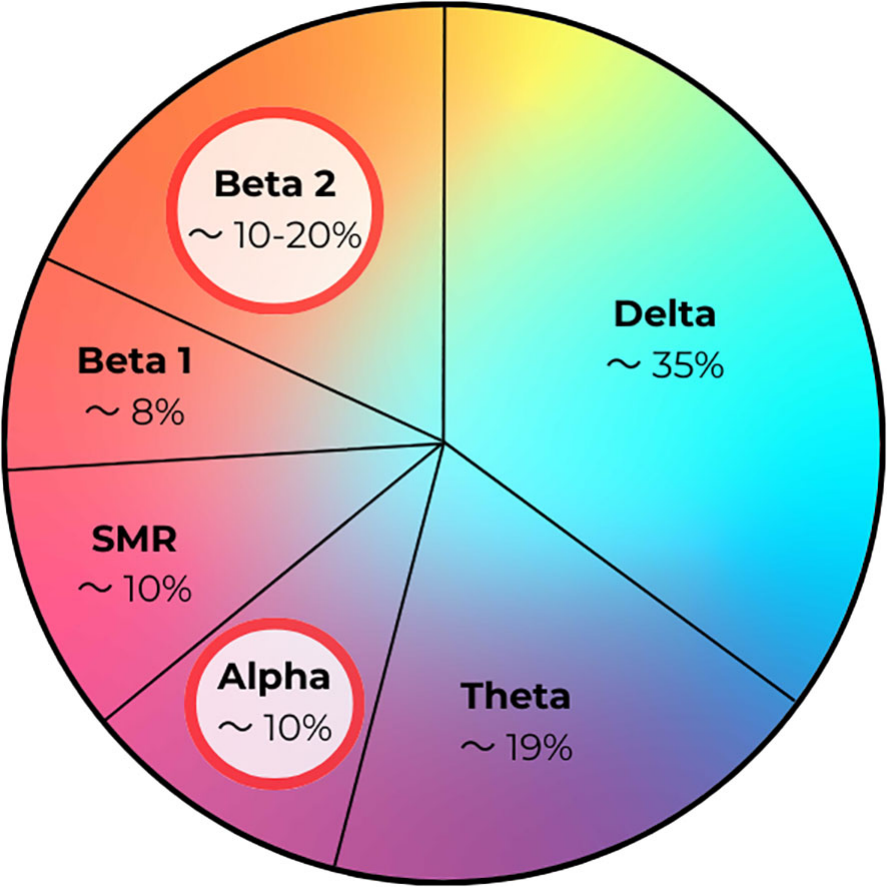
Percentage contribution of brain waves in children with autism/autism spectrum disorder (ASD).

In addition to these trends, the results of children diagnosed with autism showed subtle changes in the percentage contribution of Delta, Theta, Beta1, and SMR waves compared to the control group – **Figure 14**.

## 4 Discussion

Our study aimed to describe a brain wave pattern, potentially characteristic for children with mASD, based on QEEG analysis. The primary focus of our research was to assess changes in the amplitude of Alpha, Theta, Delta, Beta2, Beta1, and SMR waves in comparison with QEEG parameters in a control group.

Currently, the diagnosis of ASD is mainly based on observing the child’s behavior and conducting interviews with parents. This approach carries some risk of misdiagnosis, as ASD symptoms can resemble those of other neurodevelopmental disorders and may be misinterpreted by surveyed parents/caregivers (29). Rapid assessment and accurate diagnosis pose challenges for many researchers. Some suggest that measuring the brain’s bioelectrical activity may become a key avenue for developing functional biomarkers in individuals with ASD (30). Based on that, in our study, we utilized QEEG, which could serve as a valuable research tool for diagnosing ASD.

### 4.1 Delta/theta wave analysis

Analysis of the Delta and Theta frequency bands revealed statistically significant increases at the points Fz, F3, F4, Cz, C3, C4, P3, Pz, P4, T3, T4, O1 and O2. Delta waves are the slowest brain waves, occurring in the frequency range of 0.5–4 Hz. These amplitudes can be observed in patients with structural neurological damage, learning difficulties, and severe Attention Deficit Disorder. The delta rhythm can be recorded across the entire surface of the skull (15). The observation of high delta wave amplitudes in children with mASD may indicate the presence of neurobiological disturbances in this group, potentially affecting various brain functions such as sleep problems, sensory processing, and interregional communication, underscoring the need for further research into the pathophysiological mechanisms of autism (31). In our study, elevated delta wave values were recorded in all leads, which may point to the presence of ASD-related symptoms in children. This is consistent with previous studies showing that children with Level-1 ASD have higher levels of delta waves.

Similar results were provided previously: Cornew et al. performed a magnetoencephalographic study of 50 children, including 27 with ASD and 23 in the control group. Here, the children with ASD exhibited elevated absolute power of delta wave frequencies (32). Interestingly, Gabard-Durnam and colleagues also observed correlations between increased delta wave power amplitudes and the presence of ASD in the pediatric population. They discovered that a faster increase in total delta power during the first year of life differentiated infants at high risk for ASD from those at low risk. Ultimately, they showed that infants later diagnosed with ASD exhibited more dynamic growth in total delta power before the age of three, suggesting that the increase in delta power could play an essential role in predicting the risk of ASD (33). In an EEG study conducted with 27 children aged 3 to 13 years with ASD, the authors observed that patients with secondary ASD (when symptoms are associated with another known genetic condition, among others Down Syndrome or tuberous sclerosis and often involves specific somatic abnormalities) had more prominent delta frequencies compared to those with primary autism (form of ASD, where the cause is unknown), which may further suggest that increased delta waves could serve as a prognostic marker for ASD (34).

Further, Fauzan et al. presented a study describing the brainwave patterns in individuals with ASD. The authors observed differences in amplitudes between those with ASD and the control group. Similar to our research, Fauzan et al. noted general disruptions in connectivity between different lobes, with an excessive presence of slow delta waves in the frontal lobe (35). In a study using single-channel quantitative EEG to distinguish autistic children from healthy controls, the authors demonstrated that individuals with ASD had a higher relative delta and delta–alpha ratio. This study included 5 healthy subjects and 17 autistic children (36). Another piece of evidence supporting increased delta activity in children with ASD comes from a study by Stroganova et al., who analyzed absolute spectral power (SP) in boys aged 3 to 8 years. This study found a significantly higher delta power in the prefrontal region in boys with autism compared to typically developing peers. Although our research focused mainly on wave amplitude, this finding aligns with our observation of elevated delta activity in frontal regions. Notably, the authors also reported abnormal interhemispheric asymmetry, highlighting altered lateralization patterns in ASD (20). Other scientific studies have also confirmed the presence of elevated delta wave values in individuals with ASD (37–39). Another study aimed at investigating whether QEEG could indicate the severity of ASD and was conducted with 53 patients aged 3 to 12. The authors have shown that both total and regional delta power significantly increased with the severity of ASD symptoms, suggesting that elevated delta frequencies may be an indicator of mild ASD in children (40). Additionally, Pop-Jordanova et al. observed increased delta-theta spectral power in the frontal region in children with ASD. Their QEEG analysis focused on absolute power values, revealing dominant low-frequency activity (4.39 Hz) during eyes-closed recording (41). Similarly, Shephard et al. demonstrated that children with ASD + Attention Deficit and Hyperactivity Disorder (ADHD) exhibited increased delta and theta power compared to the control group (42). These studies suggest that elevated delta wave activity may have a predictive value for ASD diagnosis and follow-up.

According to canonical EEG concept, Theta waves (4–8 Hz) originate in the thalamus and limbic system. They are commonly observed when a patient is retrieving information from memory, but they are also associated with the ability to control responses to stimuli (18). Theta waves are commonly found in the frontal-central regions and are typically associated with drowsiness or heightened emotional states (43). On the other hand, high Theta wave amplitude is often associated with various emotional states, including strong nervousness and anxiety (18). Therefore, the elevated Theta waves we observed in all leads, may be related to ASD symptoms, such as difficulties with motor planning, being characteristic of individuals with autism spectrum disorders (44). Of note, also other researchers have noted elevated Theta wave amplitudes in individuals with ASD (45). E.g., in the already cited study of Cornew et al. the patients with autism spectrum disorders had greater theta power in the parietal region and along the midline (32). Moreover, several functional connectivity studies using EEG in the Theta frequency range in ASD groups have shown locally increased coherence in the frontal and temporal regions of the left hemisphere (22). Han and colleagues noted increased Theta activity, measured as elevated power, in the frontal area, the entire left hemisphere, and interhemispheric connections in children with low-functioning ASD (45). Kawasaki and colleagues recorded greater-than-normal Theta wave activity in the frontal cortex of ASD patients, measured in terms of absolute power, and found that higher Theta activity was associated with the severity of autism (46). Similarly, Chan et al. demonstrated that individuals with ASD had elevated long-range Theta coherence in the fronto-posterior area, both intra-hemispheric (in the left hemisphere) and inter-hemispheric (from the left frontal to the right posterior area) (47). In a study involving 31 children aged 2 to 5 years, diagnosed with autism, increased Theta wave frequencies were observed in the left occipital area (48). Kang and colleagues conducted an EEG study of 97 children aged 3 to 6 years, and the results showed significantly higher Theta EEG absolute power compared to the control group (49).

### 4.2 Beta wave analysis

Beta waves are among the most ambiguous brain rhythms recorded in the EEG (49). In healthy individuals with their eyes open, Beta waves were described as associated with physiological activation and play a crucial role in processes related to conscious and logical thinking, showing a tendency to stimulate these functions (39). An appropriate amount of Beta waves is essential for tasks requiring intense attention, concentration, analytical thinking, and emotional regulation. According to classic description, Beta waves can be divided into at least two main specific categories. Beta1 waves (15–20 Hz), also known as “low Beta waves,” are primarily associated with quiet, focused, introverted concentration. Opposite, Beta2 waves (20–34 Hz), known as “mid-range Beta waves,” are linked to increased energy, anxiety, and performance (17). The standard (Q-)EEG concept describes the Beta2 waves as generated in the brainstem and cerebral cortex (18).

The Beta rhythm seems to play a key role in visual and mental stimulation processes (50, 51). There is a direct cause-and-effect relationship described between Beta rhythm oscillations and the execution of motor actions (52). In the context of cognitive tasks that require sensorimotor interaction, Beta oscillations are modulated accordingly (53). This modulation is necessary for the effective processing and response to sensory stimuli, which is critical for successful interaction with the environment. Furthermore, Beta waves are characteristic of states of intense concentration, alertness, arousal, and mental activity, and are also noticeable during specific mental engagement and during the execution of motor tasks (54). In our study, patients with ASD exhibited Beta wave values exceeding established reference norms, thus showing significantly higher values compared to the control group. This pervasive increase, particularly pronounced for Beta1 (15–20 Hz) and Beta2 (20–34 Hz) activity. In general, increased Beta2 wave activity is not a desirable phenomenon (18). Excessive Beta2 waves are correlated with intense emotional tension and may suggest sensory hyperreactivity in somatosensory brain areas, as well as impulsivity and reactivity, particularly in the frontal regions, with a preponderance of the right hemisphere (55). Studies have shown the presence of structural anomalies in various brain areas in individuals with ASD, particularly in the frontal lobe region (56, 57). Dysfunction in the frontal lobes can manifest in speech delays, motor disturbances, and social communication deficits, as seen in individuals with autism spectrum disorders (58). Moreover, individuals with ASD may experience chronic stress or anxiety, which could also correlate with increased Beta2 wave amplitudes (59–61). Finally, increased Beta2 activity may also be associated with a hyperarousal state. Studies have shown that high Beta wave values (particularly in terms of increased power) are linked to high sensory processing sensitivity (SPS) (62).

The results of our study on the increase in Beta waves align with reports by Pop-Jordanova et al. In their study, they observed heightened Beta wave activity, affecting mostly the centrotemporal region, reflected in increased Beta power (41). An increase in Beta power in individuals with ASD (as compared to healthy individuals) also showed marginal significance in Orechova et al.’s experiment (63). Meanwhile, according to research conducted by Akhter et al., the most common non-epileptic anomaly in ASD patients was a varying background slowdown, followed by intermittent generalized slow high-voltage waves and increased Beta wave activity. However, this Beta wave increase was likely due to the concurrent use of antiepileptic drugs, as confirmed by their medical history (64).

In our analysis of wave distribution, we have noted an increase in percentage of Beta2 waves to about 15–20% compared to the norm, according to Sterman (15). An increase in Beta frequency in autism spectrum disorders was also reported by Coben et al. In their study, there was an increase in midline Beta power, but they also observed a reduction in absolute Beta waves over the right hemisphere in the ASD group, associated with an increase in Theta waves in that area (31).

In the study by Murias et al., individuals with ASD also showed increased Beta1 wave power compared to the control group (22). However, it is worth noting that this study focused on adults rather than children, limiting the direct comparison of our results obtained in a pediatric population.

The presence of fast rhythms in EEG is considered an electrophysiological marker of cortical activation. Therefore, an excess of Beta rhythm in the EEG of children with autism spectrum disorders could support the hypothesis of an exceptionally high state of arousal/inhibition in cortical structures in this condition (66).

However, Ewen et al. reported different results, where Beta event-related desynchronization (ERD) activity was decreased in individuals with high-functioning autism (HFA). They concluded that reduced Beta frequency in the HFA group was associated with motor skills and the severity of autism symptoms (67). These different results could be attributed to methodological limitations related to frequency band analysis, including sample size, analytical methods, or demographic data selection (68).

### 4.3 SMR/alpha wave analysis

In the frequency range of sensorimotor waves, an increase in amplitude was observed in both hemispheres compared to the control group. The sensorimotor rhythm (SMR) is a type of brain wave that occurs in the frequency range of 12–15 Hz (17). These waves originate from the ventral basal nucleus of the thalamus (18).

SMR rhythms are generated during rest and in states of low sensory attention and minimal motor activity (68). Motor activity, which suppresses SMR brain rhythm activity, can disrupt both perceptual and integrative aspects of information processing (69).

Another wave with increased amplitude is the Alpha wave. Alpha waves occur in the range of 8–12 Hz (48) and are typically observed in the posterior leads (67). Research by Klimesch et al. suggests that resting Alpha reflects active preparation of the cortical system for complex information processing. High Alpha amplitude indicates a heightened state of readiness to perform a difficult task. There are several factors influencing high Alpha amplitude, such as positive behavioral outcomes and effective inhibition of responses, as well as enhanced cognitive task performance (70). Berman et al. demonstrated that the higher the Alpha amplitude, the higher the signal-to-noise ratio (SNR) in the ASD group (71). Sutton et al. demonstrated that children with ASD exhibit different patterns of alpha wave asymmetry compared to the control group (65). Numerous studies also indicate that reduced alpha wave power may be a potential biomarker for autism (8). For example, a study by Keehn et al. reported that all children diagnosed with ASD had significantly reduced alpha wave power, which was interpreted as a manifestation of impaired cortical inhibition and ineffective stimulus processing (72).Similar results were obtained in both pediatric and adult populations, suggesting that reduced alpha activity is a stable and reproducible effect in autism spectrum disorders (22, 73). Therefore, the increased alpha activity we demonstrated in our group may indicate the existence of a different neurophysiological profile in mild forms of ASD, requiring further comparative studies, but it may also indicate the heterogeneity of this disorder. Although cortical hyperactivity (74),which is usually associated with reduced alpha wave power, has been observed in individuals with ASD, our results show that this is not true for all subgroups. This indicates that the brain in ASD may function in different ways.

Previous studies using QEEG in ASD have most often reported selective alterations in brainwave activity, such as increased delta and theta power and reduced alpha coherence, rather than global increases across all frequency bands. Our study revealed elevated frequencies of Delta, Theta, Beta1, Beta2, Alpha, and SMR waves in all examined leads in children with ASD compared to the control group. Indeed, the number of behavioral and pathophysiological features (including QEEG hallmarks) that may serve in diagnosing ASD is high. Nevertheless, our study can serve as a reference point for future researchers, aiming at declaring QEEG records as a valid biomarker for ASD workup. Additionally, our study highlights the feasibility and great ease of using QEEG in assessing autism spectrum disorders in children, suggesting that in the future, QEEG may become one of the cornerstones for ASD diagnosis.

It is worth noting that reviews of previous studies indicate that individuals with ASD most often show decreased alpha power and increased gamma power, while changes in other frequency bands are less consistent (8). Ribeiro et al. emphasized that although EEG studies consistently reveal abnormalities in ASD, the findings remain highly heterogeneous (75). Therefore, our results complement the growing body of evidence while highlighting the need for studies on larger cohorts to better define the role of QEEG as a supportive biomarker in ASD diagnostics.

### 4.4 Elevated brainwave amplitudes in ASD: implications for cortical excitability and neural development

One of the most striking findings of our study is the consistent and statistically significant increase in amplitudes across all analyzed frequency bands within the ASD group. This global pattern, rather than pointing to a dysfunction in a specific rhythm, may suggest a more fundamental dysregulation of cortical excitability. One of the leading hypotheses in the neurobiology of autism is the theory of impaired excitation-inhibition (E/I) balance, which posits a predominance of excitatory processes (76). The ‘noisier’ bioelectrical brain activity that we have observed in mASD subjects, could be an electrophysiological correlate of this phenomenon, reflecting a general state of neuronal hyperexcitability or reduced inhibitory control across various brain regions (65). Alternatively (or in conjunction with E/I imbalance) the pervasive increase in the power of slow waves (Delta, Theta) alongside an increase in the power of fast waves (Beta) may reflect the immaturity of neuronal processes and a delay in the development of cortical inhibitory mechanisms, which are crucial for information filtering and effective cognitive functioning. This widespread increase in amplitude could also be indicative of altered functional connectivity, where brain regions are either overly connected (over-connectivity) or inefficiently communicating, leading to a less efficient and more ‘noisy’ signal processing (9, 38).

Furthermore, the analysis of results under both eyes-open (EO) and eyes-closed (EC) conditions revealed a similar pattern: significantly higher amplitudes across all bands in children with ASD. This may indicate stable, endogenous differences in neuronal network organization in children with ASD (9). Minor variations in the level of amplitude increase between the EO and EC conditions warrant further, more detailed investigation.

### 4.5 Limitations of the study

It is important to acknowledge certain limitations of the present study. Firstly, while our focus on children with mASD allowed for a more specific analysis within this subset of the spectrum, the relatively small sample size ((n=24)) for this group still restricts the broad generalizability of our findings to the entire, highly heterogeneous population of children with ASD. Consequently, subgroup analyses based on varying functional levels within the broader ASD spectrum were not feasible within the scope of this study and thus were not conducted. Future research should aim to replicate these findings in larger cohorts of children with mild ASD, as well as investigate the applicability of these findings across different severity levels of ASD.

Secondly, while the 13-electrode montage employed was clinically justified and practical for use with children with ASD due to minimized preparation time, it offers lower spatial resolution compared to high-density montages (e.g., 64- or 128-channel systems). This might limit the detection of more subtle, localized brain activity patterns. Nevertheless, the use of a simplified (thus quick) method of QEEG preparation may be justified by the limited attention span of autistic children and hence the narrowed timeframe for their effective cooperation during the QEEG recording. However, the compromise that was met between the exactness of recorded data and the feasibility of the conducted records was well balanced, as we have obtained the valid sets of QEEG recordings in all included subjects.

Thirdly, despite meticulously adhering to rigorous artifact elimination procedures that incorporated both automatic algorithms and manual visual inspection, the inherent variability of EEG signals, particularly in a pediatric population with neurodevelopmental variability, consistently presents a challenge for achieving absolute signal purity. However, more advanced methods such as ICA or ASR were not used. The approach used ensured sufficient reliability of the data obtained.

Fourthly, no formal correction for multiple comparisons was applied, which may increase the risk of type I error and should be addressed in future studies. Finally, a key limitation is the lack of systematic control for potential confounding factors. The study did not include a formal assessment of the participants’ intelligence quotient (IQ), detailed data on their medication use was not collected, nor was the presence of comorbid neurodevelopmental or psychiatric conditions controlled for beyond the exclusion criteria. These variables are known factors that can influence EEG recordings.

Future research endeavors should aim to replicate these findings in larger, more homogeneous patient cohorts, potentially incorporating more advanced analytical techniques and denser electrode arrays to further refine our understanding of QEEG markers in mild ASD.

## 5 Conclusions

In our study, we have demonstrated that pediatric patients with mASD demonstrate increased amplitude in all types of QEEG waves, as well as a trend toward enhanced beta2 wave in the global QEEG spectrum. Our results are consistent with the findings of other researchers, which may indicate characteristic changes associated with autism. This confirms the reliability and reproducibility of the results and highlights the importance of QEEG as a research tool. The obtained results can be used to expand the study to a larger group of children, potentially identifying a characteristic brain wave pattern for autism in children. This may aid in better understanding the pathophysiological mechanisms of ASD.

Our study also emphasized the significance of using QEEG in assessing the presence of autism spectrum disorders in children. In the future, QEEG could become a valuable supportive tool in the assessment of ASD. The high sensitivity of this method suggests potential for detecting subtle changes in brain activity, which could support earlier identification and intervention. Early detection of ASD is crucial for improving the quality of life for patients. QEEG may help to identify characteristic brainwave patterns in children with ASD (or potentially differentiate it from other entities), which could facilitate earlier therapeutic interventions and improved developmental outcomes, enabling earlier initiation of therapy and interventions. It is worth noting that the QEEG findings in our study should be considered preliminary and require confirmation in studies with larger samples.

## Data Availability

The raw data supporting the conclusions of this article will be made available by the authors, without undue reservation.
